# Global analysis of switchgrass (*Panicum virgatum* L.) transcriptomes in response to interactive effects of drought and heat stresses

**DOI:** 10.1186/s12870-022-03477-0

**Published:** 2022-03-08

**Authors:** Rita K. Hayford, Desalegn D. Serba, Shaojun Xie, Vasudevan Ayyappan, Jyothi Thimmapuram, Malay C. Saha, Cathy H. Wu, Venu Kal Kalavacharla

**Affiliations:** 1 Molecular Genetics and Epigenomics Laboratory, College of Agriculture, Science and Technology, Delaware State University, Dover, DE USA; 2 Center for Bioinformatics and Computational Biology, Department of Computer and Information Sciences, University of Delaware, Newark, DE USA; 3 USDA-ARS, U.S. Arid Land Agricultural Research Center, Maricopa, AZ USA; 4 Bioinformatics Core, Purdue University, West Lafayette, IN USA; 5 Noble Research Institute, LLC, Ardmore, OK USA; 6 Center for Integrated Biological and Environmental Research, Delaware State University, Dover, DE USA

**Keywords:** *Panicum virgatum*, Transcriptome, Drought stress, Heat stress, Transcription factors, Gene ontology, Differential gene expression

## Abstract

**Background:**

Sustainable production of high-quality feedstock has been of great interest in bioenergy research. Despite the economic importance, high temperatures and water deficit are limiting factors for the successful cultivation of switchgrass in semi-arid areas. There are limited reports on the molecular basis of combined abiotic stress tolerance in switchgrass, particularly the combination of drought and heat stress. We used transcriptomic approaches to elucidate the changes in the response of switchgrass to drought and high temperature simultaneously.

**Results:**

We conducted solely drought treatment in switchgrass plant Alamo AP13 by withholding water after 45 days of growing. For the combination of drought and heat effect, heat treatment (35 °C/25 °C day/night) was imposed after 72 h of the initiation of drought. Samples were collected at 0 h, 72 h, 96 h, 120 h, 144 h, and 168 h after treatment imposition, total RNA was extracted, and RNA-Seq conducted. Out of a total of 32,190 genes, we identified 3912, as drought (DT) responsive genes, 2339 and 4635 as, heat (HT) and drought and heat (DTHT) responsive genes, respectively. There were 209, 106, and 220 transcription factors (TFs) differentially expressed under DT, HT and DTHT respectively. Gene ontology annotation identified the metabolic process as the significant term enriched in DTHT genes. Other biological processes identified in DTHT responsive genes included: response to water, photosynthesis, oxidation-reduction processes, and response to stress. KEGG pathway enrichment analysis on DT and DTHT responsive genes revealed that TFs and genes controlling phenylpropanoid pathways were important for individual as well as combined stress response. For example, hydroxycinnamoyl-CoA shikimate/quinate hydroxycinnamoyl transferase (HCT) from the phenylpropanoid pathway was induced by single DT and combinations of DTHT stress.

**Conclusion:**

Through RNA-Seq analysis, we have identified unique and overlapping genes in response to DT and combined DTHT stress in switchgrass. The combination of DT and HT stress may affect the photosynthetic machinery and phenylpropanoid pathway of switchgrass which negatively impacts lignin synthesis and biomass production of switchgrass. The biological function of genes identified particularly in response to DTHT stress could further be confirmed by techniques such as single point mutation or RNAi.

**Supplementary Information:**

The online version contains supplementary material available at 10.1186/s12870-022-03477-0.

## Background

Plants in the field are exposed to various environmental stresses which affect production and yield. These environmental stresses include abiotic factors such as DT, HT, and salinity and biotic stresses like pathogens, and insect pests, [1]. Abiotic stresses are reported to reduce about 50% of crop production [2]. Stress tolerance research has primarily focused on the response of plants to individual stress with limited information on plants’ adaptability to combined stresses such as HT and DT and salinity and DT [3–5]. Moreover, plants exhibit a unique expression pattern when exposed to multiple stresses [6]. Hence to bridge the knowledge gap, we have compared the transcriptional response of switchgrass when exposed to individual DT stress or a combination of DT and HT stresses.

The combined effect of DT and HT stresses has been shown to cause more damage to plants than when these stresses occur at separate times [7, 8]. The mechanisms used by plants to adapt to multiple stresses can be complex. It has been shown that the effect of one stress could have a synergistic or antagonizing effect on other stress. DT, salinity, high and low temperature have been shown to promote the occurrence of pathogens and pests [5]. In addition, the antagonizing effect of cold stress on osmotic stress during the induction of dehydration-responsive gene *RD29A* has been reported [9]. Abscisic acid (ABA) was found to antagonize jasmonate-ethylene signaling pathways and mediates defense gene expression and disease resistance in Arabidopsis [10]. Multiple stress in plants led to the expression of common overlapping genes due to a cross-talk of a signaling pathway. A previous study identified 22 genes that were induced commonly during DT, cold, and NaCl treatment [11]. Some of the molecular mechanisms adopted by plants to combat stress include the release of Heat shock proteins or chaperons that are expressed during exposure to environmental cues [12].

Transcriptome analysis of Arabidopsis showed that HT resistance is conferred by HT stress-responsive genes, plant hormones, and antioxidant enzymes [13]. The importance of transcriptional gene regulation in plants under DT and HT stresses has been previously reported [13]. RNA sequencing (RNA-Seq) has been commonly used to identify genome-wide transcript profiles in plants. Stress-responsive genes have been identified in tobacco and Arabidopsis when exposed to combined DT and HT stress by RNA-Seq technology [14, 15]. Plant responses to single stress treatment of cold, high light, salt, HT, and flagellin have been compared to various combinations of these six pair of stresses (cold and high light, salt and HT, salt and high light, HT and high light, HT and flagellin respectively). The outcome of this study revealed how plants have evolved to withstand combination of these stresses [4]. The combined effect of DT and HT stress has been studied in wheat [16]. The effect of combined abiotic stress signaling such as DT, salinity, and metal in rice was found to be complex with the involvement of multiple genes, differential expression patterns in different developmental tissues, and protein-protein interaction [17]. Furthermore, the separate impact of DT and HT and their combined effect on grain filling, physiological, vegetative, and yield traits were investigated in wheat [8].

Switchgrass (*Panicum virgatum* L.) is a C4 warm-season perennial grass identified as a potential bioenergy crop [18, 19]. It has been investigated for lignocellulosic ethanol production in the US, Canada, and Europe [20] due to its high biomass yield. It serves as a potential alternative to nonrenewable fossil fuels, thereby providing energy security sources [21]. Switchgrass requires a minimal amount of water and nutrients and can grow on marginal croplands [22]. Its rapid growth rate and broad adaptability contribute to a stable and high biomass supply. Switchgrass positively impacts the soil by improving soil quality, preventing erosion, and reducing soil nutrients [23].

Switchgrass, like many other plants, is generally faced with extreme biotic and abiotic stresses. These stresses can be detrimental by causing retardation in plant growth, development, and even death [24]. DT is a significant abiotic stress that limits switchgrass use as biofuel production. There is evidence of DT as an essential economic risk factor affecting biofuel production [25]. Molecular mechanisms underlying DT responses in plants have been addressed in various articles [26]. A previous report suggests DT could considerably reduce the yield and quality of biomass for biofuel production [27]. The effect and response of switchgrass germplasms to DT stress have been evaluated in previous studies [28–30]. High temperatures in the Southern United States are projected to reduce switchgrass biomass in 2080–2090 [22]. Similarly, various studies have reported the impact of high temperatures on switchgrass, emphasizing physiology, cell wall composition, biomass, and yield. A significant decrease in biomass yield was observed across various switchgrass genotypes due to the impact of high temperatures [22, 31]. There is increasing research in switchgrass, and among the area of research is gene regulation. Transcriptome analysis has been used to determine genes associated with biomass production in switchgrass [29]. The characterization of DT and HT responsive microRNAs has been recently reported [18]. Besides, the role of microRNAs during DT and salt stress in switchgrass has been reported [32].

Although switchgrass is an essential bioenergy crop, less information on the biology of switchgrass is available when imposed with abiotic stresses [23]. The molecular mechanisms of the tolerance of switchgrass to hot and dry climates is not well studied [18]. Therefore, understanding the effect of stress combinations in switchgrass will be important to reveal genes associated with important traits such as biomass and biofuel production in response to multiple environmental stresses. Additionally, breeding DT and HT resistant switchgrass cultivars will be an important milestone. Although several studies have reported switchgrass response to a single DT or HT stress, there are no reports as far as we know on the combination of DT and HT abiotic stresses in switchgrass, especially with prolonged exposure to DT and HT stresses.

To better understand plant responses to the full complement of environmental stresses, it is important to compare data on single stresses with data on multiple stresses. It is also important to identify the early transcriptional response to DT and HT stress versus the prolonged exposure of switchgrass to these stresses. This will provide an idea of signaling cross-talk in systems biology [33]. In this study, we used RNA-Seq approache to characterize and quantify gene expressions in response to DT and combined effects of DT and HT stresses in switchgrass.

## Results

### RNA-Seq data quality and summary

**Fig. 1:**
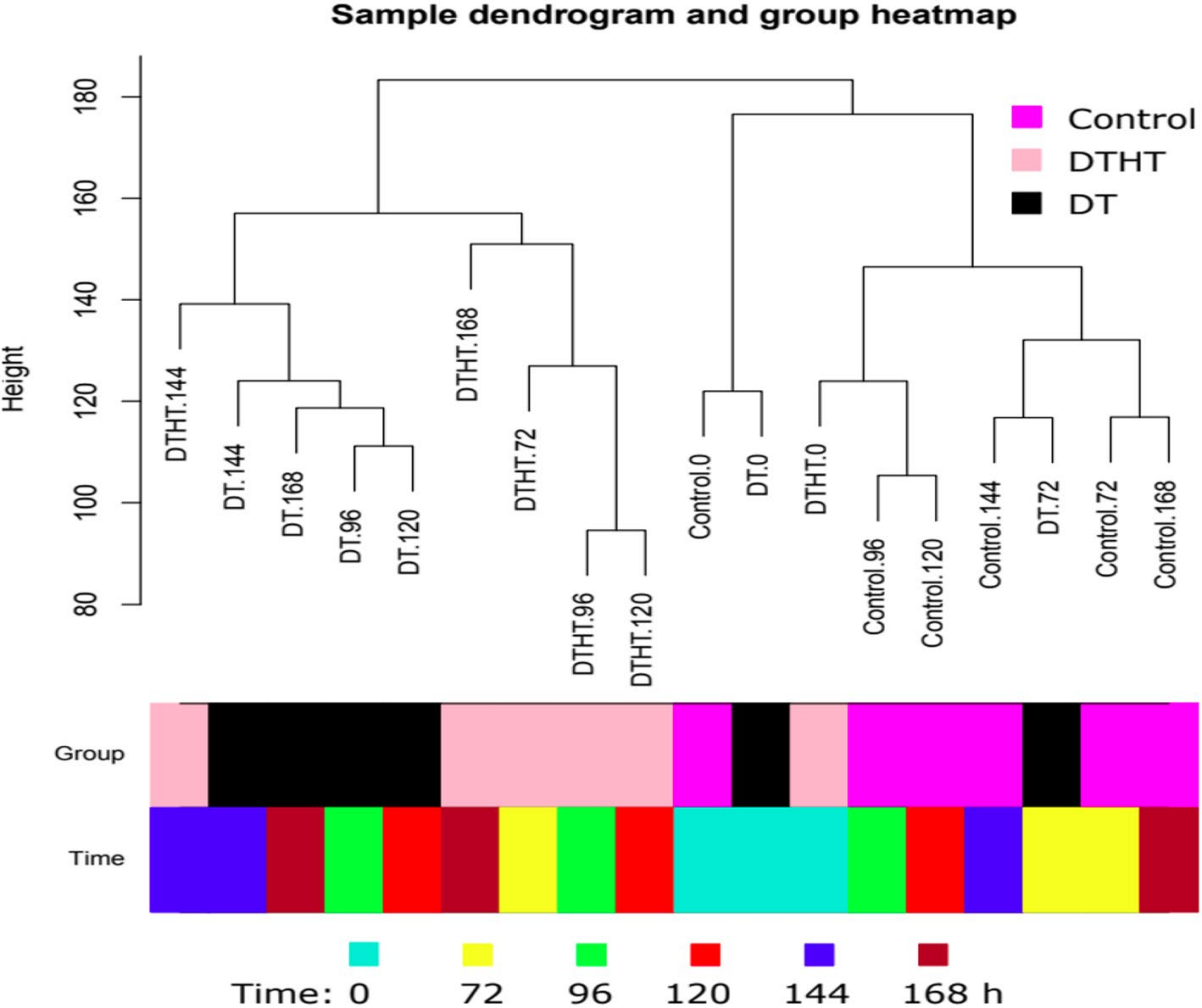
Hierarchical clustering analysis of Control, DT, and DTHT treated samples

A total of 6965 million paired-end reads were obtained from RNA-Seq samples. The number of reads in each sample was 129 million on average. Around 85% of the reads can be aligned to the reference genome. About 63% of reads were aligned to genic regions. To assess the similarities and differences among these samples, we performed a hierarchical cluster analysis of the RNA-Seq data (Fig. 1). We found that non-treated samples were grouped together except the 72 h DT treated samples. In the group of stress treated samples, DTHT samples were grouped together except 144 h DTHT sample, which clustered with the group of DT samples.

### Analysis of DT and DTHT responsive genes in switchgrass

**Fig. 2:**
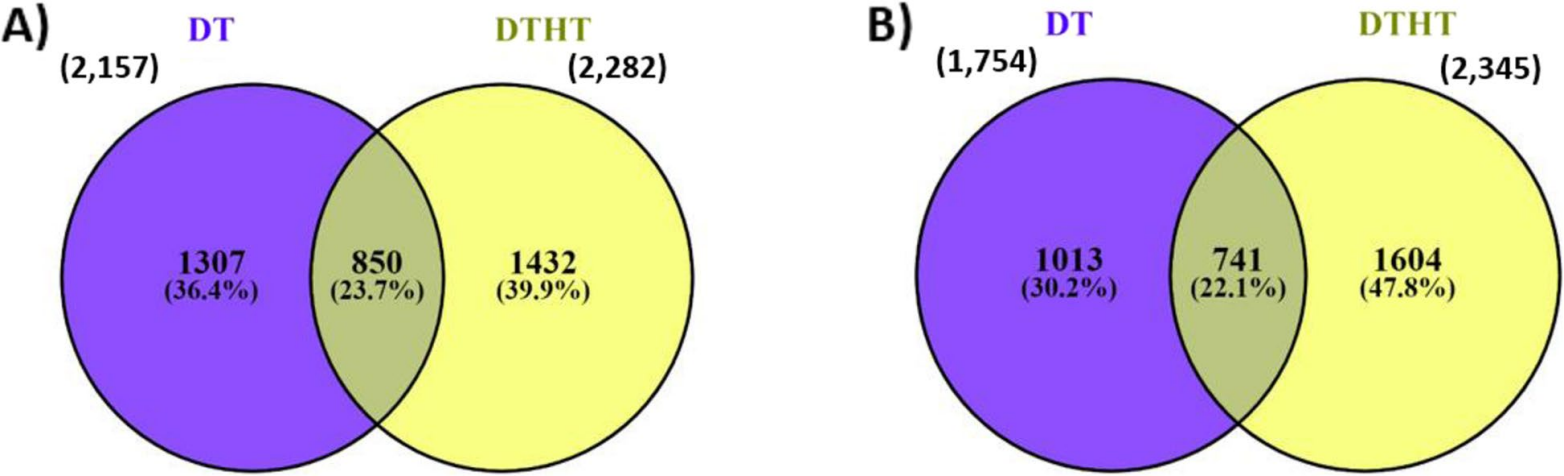
The number of common and specific up-regulated (**A**), and down-regulated (**B**) genes among switchgrass during DT and DTHT stress in the Venn diagram. The genes were significantly differentially expressed (DE) in more than one comparison of the time point, 0 h, 72 h, 96 h, 120 h, 144 h, and 168 h. DE genes for each comparison were quantified at log2 fold changes and *P*-value < 0.05

From the analysis, many genes were identified in response to the DTHT compared to only DT stress. In total, 3912 out of 32,190 genes were identified as DT and 4635 as DTHT responsive genes. Among those, 1615 genes were shared between the DT and DTHT data sets, when DT samples were compared to plants exposed to combined DTHT stress. These commonly expressed genes likely play critical roles in DT and HT tolerance in switchgrass. Further analysis showed that 1432 out of 2282 of the up-regulated responsive genes were unique (Fig. 2A) and 1604 out of 2345 down-regulated genes were unique to DTHT (Fig. 2B). Similarly, for DT samples, 1307 out of 2157 up-regulated responsive genes were unique, while 1013 out of 1754 down-regulated genes were unique (Fig. 2A and B).

In our data, Pavir.6 KG130600.v4.1 provided the best hit to Arabidopsis AT1G22360.1 (UDP-glucosyl transferase 85A2 (UGT85A2) and it is the only DT-responsive gene that showed both up and down-regulation between the time points after imposing DT treatment (Additional file 5, DT). This gene was significantly down-regulated at time points DT 96 h and DT 120 h after which its expression markedly up-regulated at 168 h.

**Fig. 3:**
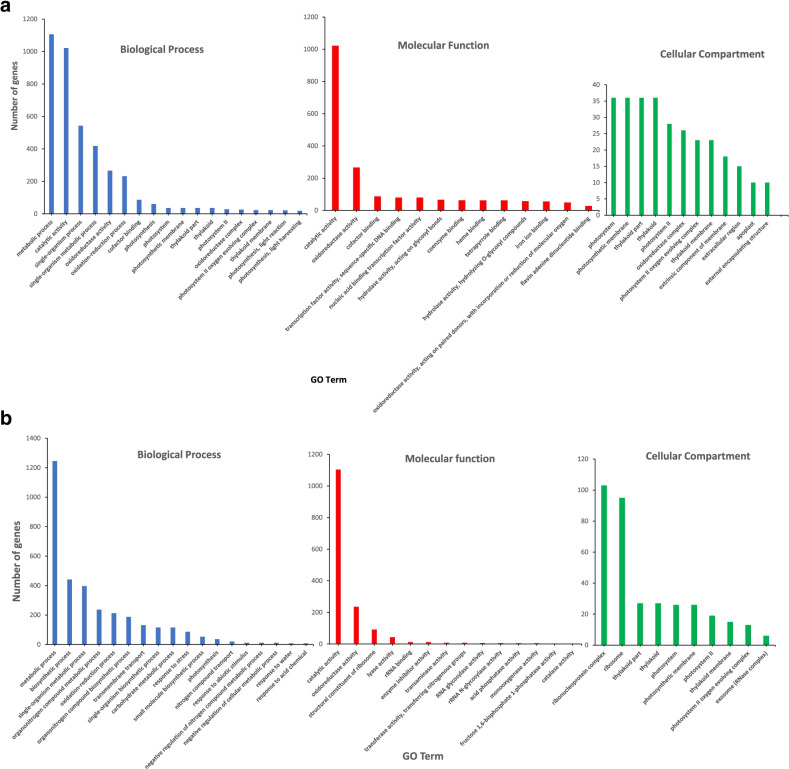
**a**. The Gene Ontology (GO) terms enriched by responsive genes to DT stress. The DEGs were annotated against the GO database. The GO terms are in the three GO domains (biological process, molecular function and cellular compartment). These terms were significantly enriched (*p* < 0.05) in combined DT and HT treated samples compared to control plants. The number of genes enriched in each term were plotted against the GO term. **b**. The Gene Ontology (GO) terms enriched by responsive genes to DTHT stress. The DEGs were annotated against the GO database. The GO terms are in the three GO domains ( biological process, molecular function, and cellular compartment). These terms were significantly enriched (*p* < 0.05) in combined DT and HT treated samples compared to control plants. The number of genes enriched in each term were plotted against the GO term

Through GO enrichment analysis (Fig. 3a, b, Additional file 6), we found that there were significantly enriched terms in all biological process, molecular function, and cellular component functional categories. In the biological process category, the enriched GO terms included photosynthesis, single-organism metabolic process, and metabolic process. GO enrichment analysis show that the GO terms; “response to stress” and “response to water”, with *p*-values (0.00042 and 0.00054, respectively) were smaller than 0.05 although the FDR values were above 0.05 (0.083 and 0.093, respectively). Eight out of 15,902 genes belonged to the GO term of response to water in the switchgrass genome whereas seven out of 3912 DT responsive genes also belonged to the GO term of response to water. In molecular function, some of the enriched terms were oxidoreductase activity, catalytic activity, and cofactor binding. In the cellular component category, the enriched terms were photosystem, photosynthetic membrane, and thylakoid part. We further performed KEGG enrichment analysis on the DT responsive genes. We found that these DEGs were enriched in the following KEGG pathways (Additional file 7): protein phosphatase 2C, glutaredoxin 3, homeobox−leucine zipper protein, jasmonate ZIM domain−containing protein, and solute carrier family, xyloglucan: xyloglucosyl transferase, HSP20 family protein, adenylate kinase, and UDP-glucuronate decarboxylase.

Pavir.9NG755000.v4.1 which provided the best hit to (ATHCHIB, B-CHI, CHI-B, HCHIB, PR-3, PR3) is a basic chitinase gene was significantly down-regulated at 144/72 h and subsequently up-regulated after prolonged DT and HT stress at 168/96 h. Similarly, genes such as Pavir.5KG627200.v4.1, Pavir.2NG348700.v4.1 and Pavir.2NG348700.v4.1 with best hit to Arabidopsis genes encoding delta 1-pyrroline-5-carboxylate synthase 2 (AT3G55610.1), cytochrome P450, family 76, subfamily C (AT2G45550.1), polypeptide 4, and DUF1012 (AT5G43745.1) respectively were significantly down-regulated at 144/72 h (Additional file 5, DTHT). These genes at 168/96 h were significantly up-regulated after prolonged DT and HT stress, suggesting the possible role of these genes in protecting the plant during extreme environmental conditions.

To study the functions of these responsive genes, GO enrichment analysis was performed. The main GO term from the enrichment analysis was the GO term (GO:0008152; metabolic process) which showed significant enrichment (FDR; 0.0014) (Fig. 3). None of the GO terms shows significant enrichment in combined DT and HT stress responsive genes, indicating that DTHT transcriptomic changes were not predictable from single stress treatments. In the category of biological process, there were 10 most enriched GO terms with *P*-value <= 0.05. These 10 GO terms were response to water, single-organism metabolic process, single-organism biosynthetic process, response to abiotic stimulus, organonitrogen compound metabolic process, photosynthesis, oxidation-reduction process, response to stress, nitrogen compound transport, and transmembrane transport respectively. We further performed KEGG enrichment analysis on the DTHT responsive genes (4635 genes). We found that these responsive genes were enriched in the following KEGG pathways (Additional File 7): adenylate kinase and protein phosphatase 2C.

### HT responsive genes in switchgrass

The HT stress genes were deduced from the DEGs of DT and DTHT. In total, 2338 out of 32,190 genes were identified as HT responsive genes (Additional file 5). There were 1064 up-regulated genes and 1274 down-regulated genes. The functions of these responsive genes and GO annotation were presented (Additional file 6). In the category of biological process, these genes showed enrichment in the GO terms of organic cyclic compound catabolic process, organonitrogen compound catabolic process and heterocycle catabolic process, etc. In the category of molecular function, these genes showed enrichment in the GO terms of organic cyclic compound catabolic process, organonitrogen compound catabolic process and heterocycle catabolic process, etc. In the categories of cellular components, these genes showed enrichment in the GO terms of photosystem II oxygen-evolving complex, photosystem II, and thylakoid membrane. We also performed KEGG enrichment analysis on the HT specific responsive genes. We found that these responsive genes were enriched in the jasmonate ZIM domain-containing protein pathway (Additional File 7).

### Transcription Factors (TF) for DT, DTHT and HT responses

**Fig. 4:**
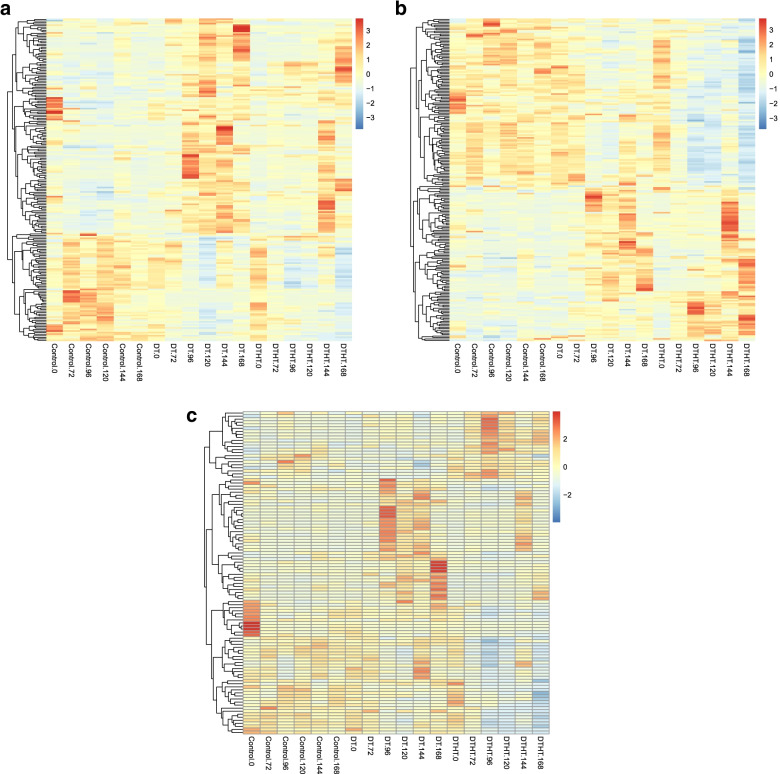
Heat map with clusters based on FPKM values for **A**) DT vs Control, **B**) DTHT vs control and **C**) DTHT vs DT TFs. The Heat map shows a grouping of control samples and stress samples. Extended periods of DTHT to stress samples showed abundant up-regulated TFs (**A** and **B**) and down-regulated TFs (**C**) compared to their control samples. For example, there were more responsive TFs which were up-regulated at time 144/72 h compared to its control sample at Control 144/72 h (**A**)

The TFs identified from the analysis are shown in Table 1, and Additional file 8. These DT and DTHT responsive TFs belong to 45 different TF families. Out of 91,838 proteins on the switchgrass genome, 3948 were identified as transcription factors (TFs). A total of 1383 TFs were identified out of 32,190 genes that were used for identifying stress responsive genes. There were 209 genes identified as TFs out of 3912 DT responsive genes. Heat maps were generated to show expression patterns of these 209 genes in all the samples (Fig. 4A). Similarly, there were 220 genes identified as TFs out of 4635 DTHT responsive genes. A heat map was generated to show expression patterns of these 220 genes in all the samples (Fig. 4). A total of 106 genes out of the 2339 predicted HT responsive genes, were identified as TFs. Heat map was generated to show expression patterns of these 106 genes in all the samples (Fig. 4C).

**Table 1:** Different families of TFs responsive to solely DT and combined DTHT stresses

Transcription factor type	DTvsCtrl	DTHTvsCtrl	DTHTvsDT
bHLH	22	20	10
NAC	16	15	13
ERF	19	19	6
bZIP	17	17	5
MYB_related	10	17	10
MYB	12	15	7
WRKY	14	11	6
HD-ZIP	15	7	3
C3H	7	13	1

### Pathway analysis of DT and HT responsive genes

**Fig. 5:**
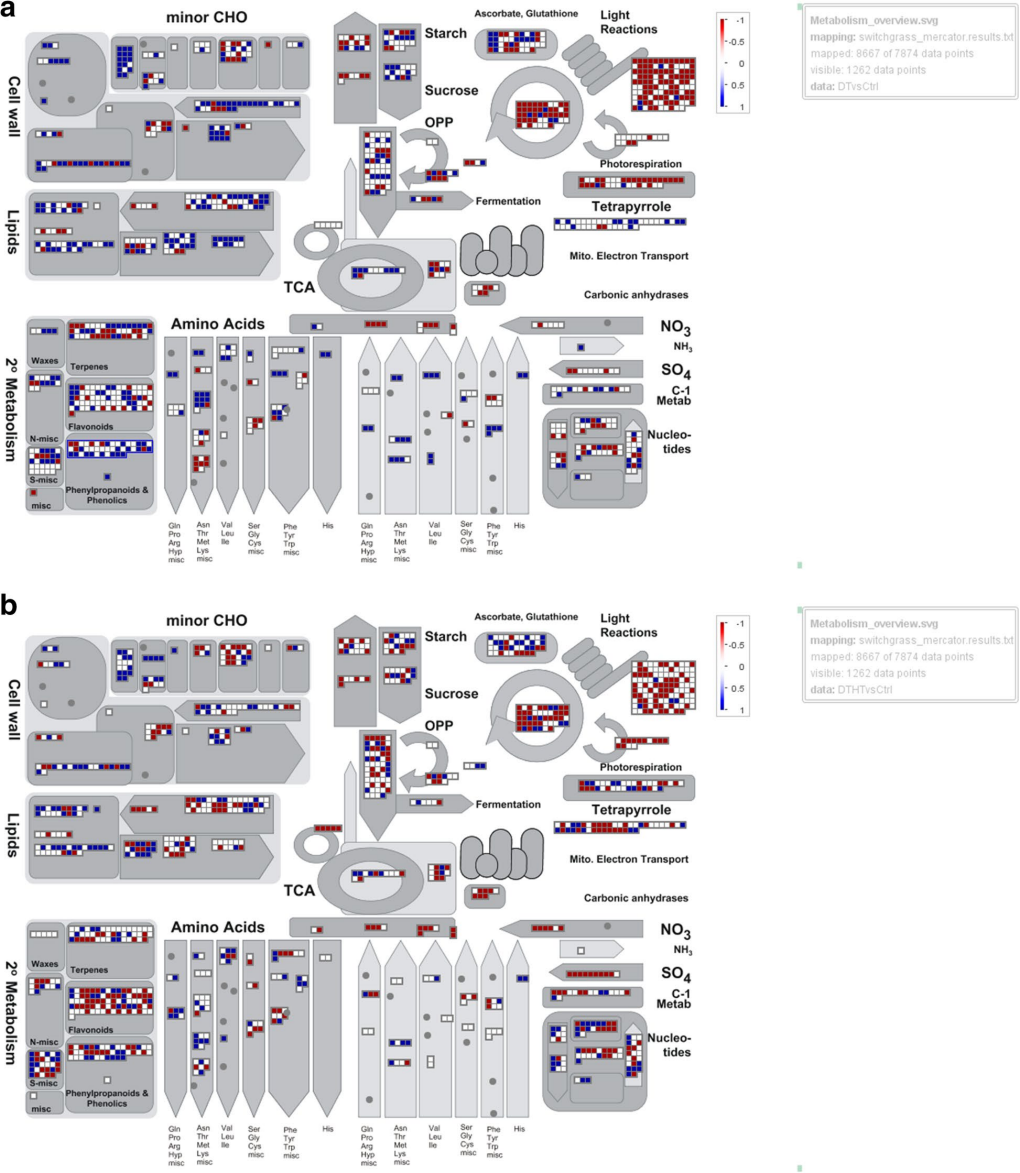
Metabolism overview in MapMan showing the DEGs between DT vs Control (**A**) and DTHT vs control (**B**) switchgrass samples. The log-fold ratio is indicated as a gradient with red color (down-regulated) and blue color (up-regulation)

An overview of the secondary metabolism pathway is displayed in Fig. 5(A and B). We found a large number of plant secondary metabolites such as flavonoids, terpenes, and phenylpropanoids were down-regulated in DTHT vs control samples compared to DT vs control samples.

### Co-expression network

**Fig. 6:**
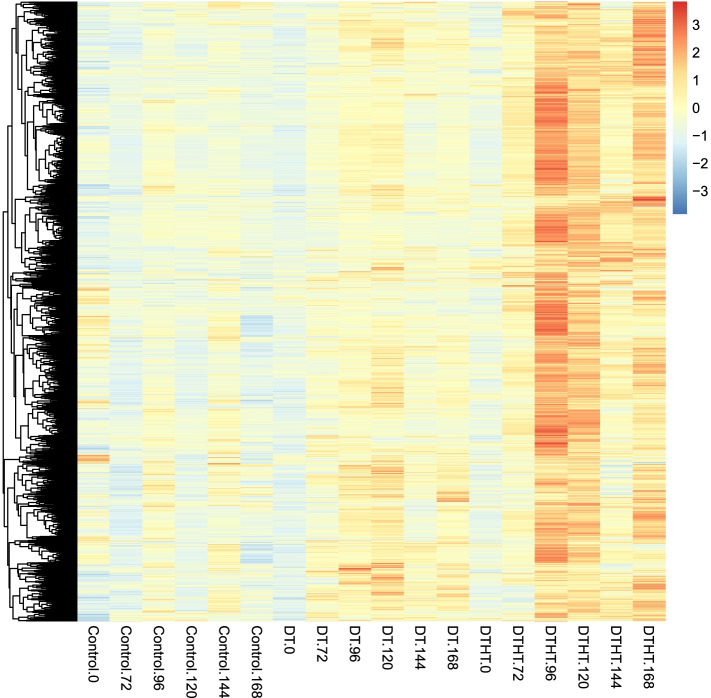
Heat map indicating genes enriched in module 1 from the WGCNA analysis. DTHT and HT responsive genes were enriched in module 1

We performed weighted gene co-expression network analysis to identify genes involved in response to the DT and DTHT stresses. Most of co-expressed genes usually participate in the same biological processes [34–36]. In our co-expression analysis we identified 68 modules with distinct expression patterns (Additional file 11). To study whether the DEGs were enriched in some of the modules, Fisher’s exact test and multiple test correction (Benjamini-Hochberg method) were performed [4] . Of the modules that have more than 100 genes, DT responsive genes were enriched in module 5, 7, 14, 17 and 25. DTHT responsive genes were enriched in module 1, 2, 3, 7 and 17. HT responsive genes were enriched in module 1, 2, 8, 9, 15, 16, 17 and 25. GO enrichment analysis of the genes of these modules were performed using agriGO. Results for GO enrichment are provided in (Additional file 12). Heat maps were generated for these 12 unique modules (Additional file 2). In module 7 and module 17, both DT responsive genes and DTHT responsive genes were enriched. In module 7 and module 17, genes were up-regulated after stress treatment. In module 7, the genes were enriched in GO terms of response to water, response to acid chemical, lipid biosynthetic process, and response to the oxygen-containing compound, or biological process. In module 17, the genes were enriched in GO terms of regulation of nucleic acid-templated transcription, regulation of RNA biosynthetic process, regulation of RNA metabolic process and regulation of transcription, DNA-templated, etc. for biological process. In module 1 (Fig. 6), most genes were up-regulated during the initial HT treatment at DTHT 96/24 h. Down-regulation of most of the genes in the same module occur and then up-regulated again at an extensive HT at DTHT168/96 h. Similarly, in module 8 which is enriched with HT responsive genes, showed upregulation of genes at the initial stage of imposing HT at DTHT96/24 h. In module 1, the genes were enriched in GO term biological processes such as translation, peptide biosynthetic process, amide biosynthetic process and peptide metabolic process. In module 8, the genes are enriched in GO terms including; multi-organism reproductive process, multi-multicellular organism process, cell recognition, and pollination for biological processes. Also, DTHT responsive genes were enriched in module 9 with most of the responsive genes recorded at time point DTHT96/24 h and DTHT120/48 h. A number of the genes recorded at DTHT96/24 h and DTHT120/48 h were enriched in different class of metabolic processes.

### DT and DTHT responsive genes in DroughtDB

There were 386 genes from the switchgrass genome that have the best hits to Arabidopsis genes in the droughtDB [37] Of these 386 genes, 172 were found in the 32,190 genes in this study. Detailed gene expression patterns of these 172 genes were shown in the heat map (Additional file 3). Out of these 172 genes, there were 35 DT responsive genes and 27 DTHT responsive genes in which 12 were common (Additional file 13). A list of the DT and DTHT genes have been indicated in Tables 2 and 3, respectively. The gene IDs, biological functions, the phenotype of mutants, references, tags of the genes from Arabidopsis can be obtained. For example, three genes are described in detail which play an important role in DT response: Pavir.1KG544600.v4.1 is homologous to KAT2 in Arabidopsis. In Arabidopsis, the *kat2–3* mutant shows ABA-insensitive phenotypes and KAT2-overexpressing transgenic lines show strong ABA-hypersensitive phenotypes (ABA-induced stomatal closure and inhibition of stomatal opening) [26]. In our data, Pavir.1KG544600.v4.1 showed increased gene expression levels under both DT and DTHT treatments. In Arabidopsis, HAB1/PP2C is known as a major negative regulator of ABA signaling and its mutant shows hypersensitive to ABA [38]. In our data, Pavir.8NG117400.v4.1, homologous to HAB1/PP2C, showed increased gene expression level under both DT and DTHT treatments. Additionally, the ABCG22 (Pavir.9NG742000.v4.1) from Arabidopsis is an ABC-transporter and a knockout of ABCG22 caused Arabidopsis to be more susceptible to DT stress [39]. From our data Pavir.9NG742000.v4.1 showed increased gene expression level under both DT and DTHT treatment. The 386 switchgrass genes with best hits to Arabidopsis genes in droughtDB were used to generate the heat map (Additional file 3).

**Table 2:** List of DT-responsive genes identified in switchgrass in the droughtDB

Gene id	Gene	Biological Function
Pavir.9NG610900.v4.1	GolS1	Galactinol Synthase, catalyzes the first step in the biosynthesis of Raffinose Family Oligosaccharides (RFOs) from UDP-galactose
Pavir.6NG274900.v4.1	AREB1	bZIP TF, ABRE binding
Pavir.6KG307800.v4.1	ABF4	ABA responsive element (ABRE) binding bZIP factor
Pavir.5KG406700.v4.1	ABCG40	ABC-transporter, ABA import
Pavir.2KG548500.v4.1	OST1/SRK2E	Kinase-like (open stomata 1), activated by ABA, activates SLAC1
Pavir.2NG401700.v4.1	ATHB6	homeodomain protein, target of ABI1
Pavir.2NG618000.v4.1	GolS2	Galactinol Synthase, catalyzes the first step in the biosynthesis of Raffinose Family Oligosaccharides (RFOs) from UDP-galactose
Pavir.9KG306600.v4.1	GSTU17	glutathion s-transferase U17
Pavir.2NG248100.v4.1	MYB44	MYB type TF
Pavir.7KG296100.v4.1	AGO1	Argonaute1
Pavir.9KG354500.v4.1	MYC2	transcriptional activator of ABA signaling
Pavir.4KG090000.v4.1	AVP1	vacuolar membrane H + -Pyrophosphatase
Pavir.9KG421700.v4.1	GolS1	Galactinol Synthase, catalyzes the first step in the biosynthesis of Raffinose Family Oligosaccharides (RFOs) from UDP-galactose
Pavir.6KG279400.v4.1	FAD8	fatty acid desaturase
Pavir.1KG544600.v4.1	KAT2	3-ketoacyl-CoA thiloase-2
Pavir.1KG312700.v4.1	ERD1	chloroplast-targeted Clp protease reg SU
Pavir.3KG112200.v4.1	DHAR2	dehydroascorbate reductase
Pavir.6NG207900.v4.1	XERICO	small protein, N-term- TM domain and RING-H2 zinc-finger motif
Pavir.1NG392600.v4.1	PIP1;4	PIP
Pavir.1NG081300.v4.1	AVP1	vacuolar membrane H + -Pyrophosphatase
Pavir.8NG117400.v4.1	HAB1	PP2C
Pavir.6KG334900.v4.1	XERICO	small protein, N-term- TM domain and RING-H2 zinc-finger motif
Pavir.2KG570400.v4.1	GolS2	Galactinol Synthase, catalyzes the first step in the biosynthesis of Raffinose Family Oligosaccharides (RFOs) from UDP-galactose
Pavir.5KG405500.v4.1	HAB1	PP2C
Pavir.9KG536300.v4.1	SQE1	squalene epoxidase1
Pavir.J678200.v4.1	AAO3	Arabidopsis aldehyde oxidase, catalyzes final step in ABA biosynthesis
Pavir.9KG308600.v4.1	GSTU17	glutathion s-transferase U17

**Table 3:** List of genes responsive to combined DT and HT stress in switchgrass from the droughtDB

Gene_id	Gene	Biological Function
Pavir.9NG211300.v4.1	ABO1/ELO1	subunit of Elongator, a multifunctional complex with roles in transcription elongation, secretion and tRNA modification
Pavir.9NG493600.v4.1	GSTU17	glutathion s-transferase U17
Pavir.2NG618000.v4.1	GolS2	Galactinol Synthase, catalyzes the first step in the biosynthesis of Raffinose Family Oligosaccharides (RFOs) from UDP-galactose
Pavir.5NG017000.v4.1	SLAH3	guard cell S-type anion channel (SLAC1 homolog)
Pavir.7KG296100.v4.1	AGO1	Argonaute1
Pavir.1NG551600.v4.1	PIP1;4	PIP
Pavir.9NG671400.v4.1	PEPCK	PEP carboxykinase
Pavir.6KG279400.v4.1	FAD8	fatty acid desaturase
Pavir.9KG480900.v4.1	APX2	Ascorbate peroxidase 2, H2O2 scavenger
Pavir.1KG544600.v4.1	KAT2	3-ketoacyl-CoA thiloase-2
Pavir.1KG312700.v4.1	ERD1	chloroplast-targeted Clp protease reg SU
Pavir.3KG112200.v4.1	DHAR2	dehydroascorbate reductase
Pavir.1NG545200.v4.1	AGO1	Argonaute1
Pavir.9NG719800.v4.1	GPA1	alpha subunit of heterotrimeric GTP-binding protein
Pavir.J075500.v4.1	AAO3	Arabidopsis aldehyde oxidase, catalyzes final step in ABA biosynthesis
Pavir.9KG118700.v4.1	GSTU17	glutathion s-transferase U17
Pavir.1NG081300.v4.1	AVP1	vacuolar membrane H + -Pyrophosphatase
Pavir.8NG117400.v4.1	HAB1	PP2C
Pavir.9NG671500.v4.1	PEPCK	PEP carboxykinase
Pavir.6KG334900.v4.1	XERICO	small protein, N-term- TM domain and RING-H2 zinc-finger motif
Pavir.2KG570400.v4.1	GolS2	Galactinol Synthase, catalyzes the first step in the biosynthesis of Raffinose Family Oligosaccharides (RFOs) from UDP-galactose
Pavir.7NG063700.v4.1	MRP4	multidrug resistance-associated protein, ABC transporter
Pavir.9KG517100.v4.1	PEPCK	PEP carboxykinase
Pavir.3KG456000.v4.1	ERD1	chloroplast-targeted Clp protease reg SU
Pavir.6NG268500.v4.1	XERICO	small protein, N-term- TM domain and RING-H2 zinc-finger motif
Pavir.7KG292400.v4.1	CBF4	DREB family TF
Pavir.2KG247300.v4.1	PARP1	poly(ADP-ribose) polymerase

### Validation of RNA-Seq results using qRT-PCR

**Fig. 7:**
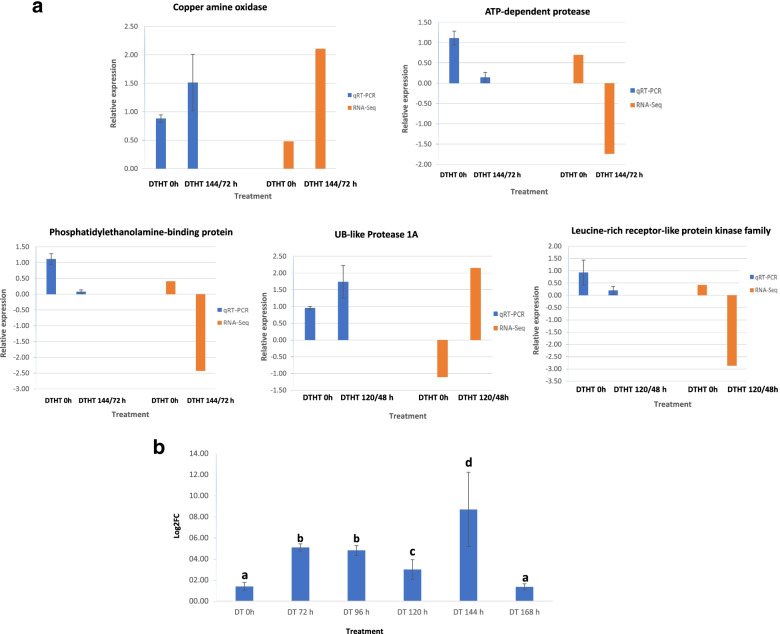
**a**. Validation of the relative expression levels of five selected genes responsive to combined DTHT stress from RNA-Seq analysis by quantitative real-time PCR (qPCR). The genes selected were differentially expressed, and the time point at which these genes showed high expression from the RNA-Seq data were selected with its control for qPCR validation. **b**. Validation of relative expression of DT-responsive gene UDP-glucosyl transferase 85A3. UDP-glucosyl transferase 85A3 was up-regulated and down-regulated at different time points during DT stress from the RNA-Seq data. The expression pattern of the qPCR analysis is like results from the RNA-Seq analysis. The different alphabets in the Figure show that the samples collected from the different time point of DT are significantly different from the control at *p*-value< 0.05. qPCR results from two technical replicates and three biological replicates were analyzed using ANOVA from Minitab 18 software. The x-axis shows the treatment imposed on switchgrass. The y-axis shows the relative expression of the genes

Seven candidate genes responsive to DTHT stress were selected from the RNA-Seq data for validation by performing qRT-PCR (Fig. 7A and B). The expression pattern of the selected genes was consistent with the RNA-Seq results.

## Discussion

DT or HT stress alone has been found to affect switchgrass physiology and cause a reduction in biomass yield [22, 29]. Extensive reports on transcriptome changes in plants during DT stress have been reported in both plant models and crop species [40]. The transcriptional response of switchgrass when imposed with solely DT or HT stress has been reported in previous studies [22, 29, 30]. However, transcriptome data associated with switchgrass when imposed with the combination of DTHT are not available. Molecular mechanisms during DTHT in plants such as lentil, cereals, and Kentucky bluegrass [41–43] have been reported. The primary objective of this study was to understand the transcriptional changes and molecular mechanisms in switchgrass in response to DT and the combined effects of DTHT.

### Genes differentially expressed due to solely DT stress

In this study, water deficit in switchgrass triggered an up-regulation of more genes than down-regulated genes (Fig. 2). One of the DT-responsive genes identified from our analysis (Pavir.9KG421700.v4.1) and reported in the droughtDB is galactinol synthase (Gols1). Gols1 catalyzes the biosynthesis of raffinose family oligosaccharides (RFOs). The RFO biosynthetic pathway is a major metabolic activity in plants and has been found to respond to various abiotic stresses. RFOs have emanated as essential molecules in plants during stress due to their antioxidant and membrane stabilizing properties. RFOs can be found in the chloroplast, which indicates its role in regulating genes in the photosystem II pathway [44, 45]. Among DT-responsive genes that were shown to be induced in our analysis is OST1 (Pavir.9KG103200.v4.1). OST1 is found in stomatal guard cells and is known to activate SLAC1 which is required for stomatal closure during DT in plants [46]. DT stress activates the production of the hormone ABA. Mustilli et al. [47] reported ABA-induced stomatal closure, which is impaired in *ost1*.

AREB1 (Pavir.J643700.v4.1) was also identified as a DT-responsive gene from our analysis and in the droughtDB (Table 2). It has also been found that the AREB subfamily of proteins and orthologues of AREB are found to be involved in ABA signaled transduction [48]. ABA plays an important role in plants and is involved in various physiological and developmental processes, including stomatal closure and response to a myriad of abiotic stresses such as cold, DT, and salinity [49]. Targets of ABA-dependent pathways recruit transcription factors such as AREB at the promoter sites to activate transcription. During DT stress, the level of ABA increases, causing ABA receptors PYR/PYL/RCAR to recruit phosphatase PP2C (identified in the KEGG pathway analysis in Tables 1 and 2) for downstream activation in the ABA-dependent signaling pathway [50]. ABA is known to regulate a large number of dehydration-responsive genes, which is associated with DT tolerance. These genes are not limited to late embryogenesis abundant (LEA), Responsive to ABA 18 (RAB18), and RD22. Apart from the ABA-dependent genes, other DT-responsive genes are also ABA-independent. An example of an ABA-independent gene belongs to the family of dehydration-responsive element-binding (DREB) protein. DREB2 was up-regulated in the switchgrass plants imposed with DT. In various studies, DREB is more involved in DT stress and has been identified in rice and maize [30]. As expected, LEA, RD22, and RAB18 were induced with DT stress from our study. There were 35 DT responsive genes and 27 DTHT responsive genes with 12 overlapping genes in the droughtDB. Some of the genes identified as DT-responsive from our study have been listed in the manually curated compilation of molecularly characterized genes that are involved in DT stress response (Tables 2 and 3). These genes include AREB/ABF and glutathione S-transferases (GSTs). Previous reports indicates that overexpression of ABF4/AREB2 lead to ABA-hypersensitive phenotypes in Arabidopsis. Similarly, transgenic Arabidopsis plants with enhanced AREB/ABF expression showed enhancement in DT tolerance, indicating the role of AREB/ABF in ABA response and stress tolerance [48, 51]. GSTs have been reported to a significant role in oxidative stress metabolism. Glutathione S-transferase U17A (GSTU17) is among the genes identified in the switchgrass samples under DT stress. In another study, mutants of GSTU17 in Arabidopsis became more tolerant to DT stress and salt stress than wild-type plants suggesting the role of GSTU17 in DT and salt stress tolerance [52].

Photosynthesis is among the processes affected by plant dehydration. In response to the waterdeficit in the switchgrass plants, transcripts encoding Rubisco activase, Rubisco methyltransferase family protein, photosystem II subunit O-2 (PSII), phosphoenolpyruvate carboxylase family protein initiation of CO_2_ into oxaloacetate in C4 plants [53] and phosphoenolpyruvate carboxylase: encoded by Ppc genes for initial fixation of CO2 were down-regulated. Two genes, carbonic anhydrase (associated with carbon-fixing and metabolism in C4 plants) and phosphoenolpyruvate carboxykinase 1 which was previously identified by Ayyappan et al.  [54] as a C4 photosynthetic enzyme were down-regulated in response to the DT stress. These findings are consistent with a report on the down-regulation of genes associated with photosynthesis during abiotic stress. Interestingly, we saw in our analysis that another transcript, Pavir.4NG244100.v4.1annotated as photosystem II subunit P-1 was down-regulated. Down-regulation of PSII affects electron transport, leading to the generation of harmful reactive oxygen species (ROS). A controlled amount of ROS protects the plant from DT as part of the signaling (ABA-dependent) pathways. However, an excessive amount of ROS which can be produced due to prolong DT could destroy critical cellular machinery of the plant while under DT stress [55]. From our analysis, Pavir.6NG292200.v4.1 annotated as Fe superoxide dismutase 3, and Pavir.3KG389500.v4.1, annotated as manganese superoxide dismutase 1 were up-regulated as scavengers of ROS to enhance the antioxidant defense of the plants under DT stress. In a previous study, the expression of Mn-SOD in transgenic *Medicago sativa* (alfalfa) plants showed increased tolerance against DT injury.

Similarly, alfalfa’s in cold conditions showed an increased expression of Mn-SOD and Fe-SOD [56, 57]. Understanding the antioxidant defense pathway will help to enhance switchgrass under DT stress. It is interesting to note that from our analysis Pavir.1KG123700.v4.1 annotated as 3-ketoacyl-CoA synthase 11 was up-regulated at four different time points of DT conditions. A recent study shows that 3-ketoacyl-CoA synthase (involved in lignin biosynthesis) could help to improve DT tolerance in tea plants [58]. Similarly, Pavir.9NG554400.v4.1 annotated as basic helix-loop-helix (bHLH) DNA-binding superfamily protein was down-regulated at four different time points of DT. Waseem et al. (2019) showed that overexpression of bHLH enhanced abiotic stress tolerance in tomatoes [59]. These genes could provide insight in providing DT tolerance in switchgrass especially during prolonged exposure to DT.

KEGG pathway enrichment results showed that 12 genes were enriched in the term glutaredoxins. Glutaredoxins have been shown to be involved in different stress responses and regulation of the Krebs cycle and signaling pathways. Overexpression of some members of the glutaredoxin family modulated plant response to various stresses. For example, transgenic tomato plants with overexpression of SIGRX1 exhibited tolerance to hydrogen peroxide, DT, and salt stress [60]. One of the significant pathways enriched by the DT-responsive genes from this report was response to water. Another report by Bhardwaj et al. (2015) identified GO terms for DT *Brassica juncea* samples which include response to water deprivation (GO:0009414) [61].

### Genes differentially expressed due to DTHT stress

From our analysis, most of the genes in response to combined DTHT were down-regulated (Fig. 2). A combination of DTHT stress in Arabidopsis caused up-regulation of more transcripts compared to down-regulated transcripts, although this is in contrast to our findings [15]. In another report, several abiotic stress factors not limited to DT and HT stress led to down-regulation of multiple genes, indicating general transcriptional repression [62]. The transcriptome responses of the control switchgrass plant and those subjected to individual DT and combination of DTHT stress were different. However, there were common DEGs in response to DT stress and a combination of DTHT stress. A significant overlap of transcripts expressed in DT or HT stress and combination of DTHT was found in plants in response to cold, DT, HT, and salt stress [11, 15]. A similar finding was observed in tomato cultivars exposed to individual DT stress and combined DTHT. Single DT treatment on tomato cultivars had a considerable effect on HT stress [63]. This finding could explain why more genes responsive to DT were identified in combined DTHT stress plants. Jia et al. [64] identified an overlap of genes such as those involved in hormone metabolism (ABA) in *Populus simonii* when a single DT or HT was compared to combined DT and HT stress. The overlap suggests specific defense mechanisms by plants in response to abiotic stresses, which can be further explored. We identified 35 DT and 27 DTHT responsive genes in switchgrass, of which 12 were common between the two conditions. The key genes that played an important role in switchgrass performance under DT and DTHT include RFO, OST1, AREB1, GSTU17. Open Stomata 1 (OST1) is involved in the ABA regulation of stomatal response ([65]. RFO is a biosynthetic pathway, and it’s involved in a major metabolic activity in plants and has been found to respond to various abiotic stresses [44] . AREB1 is a transcriptional activator, and it controls the ABA signaling to improve DT tolerance [66]. Documentation of the response of GSTs to a plethora of environmental stress responses has also been documented. GSTU17 in Arabidopsis was seen to provide DT and salt stress tolerance [67, 68]. This finding suggests the possible expression of GSTU17 in both DT and DTHT samples. Most of the genes were revealed in the droughtDB (Tables 2 and 3).

In response to both DTHT, factors such as LEA and Heat shock proteins (HSPs) were up-regulated in our analysis. LEA and HSPs have been reported as responsive to DT and extreme temperatures, and they play an essential role in protecting the plant during stress. Wang et al. [69] reported the response of LEA and HSPs to DT, salinity, and HT stress. Interestingly, Pavir.5KG018400.v4.1 (LEA14) was significantly up-regulated at 168 h. The same transcript was up-regulated at time point 168/96 h in both DT and HT-treated samples. LEA proteins accumulate primarily in plants during water deprivation. However, LEA proteins have been reported to respond to extreme temperatures as well. A previous report in *Brassica juncea* indicated that LEA showed a 40-fold increase during DT stress and a 10-fold increase in HT stress [61]. This finding suggests that LEA14 could be a candidate gene for breeding in areas with severe DT and extreme temperatures.

We identified several Heat shock proteins (HSPs) in the switchgrass samples imposed with DTHT stress. Pavir.9NG640000.v4.1 and Pavir.9KG490200.v4.1 transcripts annotated as HT-shock protein 70 T-2 and Heat shock protein-70 respectively were significantly up-regulated at four different time points of the study. Other HSPs identified include Pavir.1NG519200.v4.1 (HSP20-like chaperones superfamily protein), Pavir.1KG194500.v4.1 (Heat shock protein 17.6A), Pavir.9NG570500.v4.1 (Heat shock protein 21), Pavir.6KG320100.v4.1 (Chaperone protein htpG family protein), Pavir.9KG212600.v4.1 (Heat shock protein 60). In a previous study, Grigorova et al. (2011) observed the induction of HSPs in wheat samples imposed with DTHT stress compared to single DT stress [16].

Additionally, Pavir.9KG480900.v4.1 annotated as ascorbate peroxidase 1 (APX) and Pavir.7KG159800.v4.1 (stromal ascorbate peroxidase) were also found to be up-regulated by our analysis. The role of the *APX* gene in response to abiotic stress conditions such as temperature, high light, DT, salinity, and heavy metals has been reported [70].

The Pavir.9NG211300v4.1 transcript encoding the ABO1/ELO2 gene was identified in the DroughtDB and only responsive to DTHT stress. ABO1/ELO2 is an ABA-induced gene, and mutants showed affected development of guard cells, causing a decrease in the number of stomatal cells. ABO1/ELO2 is a subunit of Elongator, a multifunctional complex with roles in transcription which provided an uncommon mechanism of DT tolerance in Arabidopsis [71]. From our analysis, ABO1/ELO2 was up-regulated and this could be in response to the combined effect of DTHT to induce ABA hormones to regulate the stomata cells. Interestingly, another transcript Pavir.2KG247300.v4.1 codes for poly(ADP-ribose) polymerase (PARP1) were responsive in only DT and HT-treated switchgrass samples. PARP regulates transcription, metabolism and is involved in organizing the chromatin structure. Also, PARP responds to both biotic and abiotic stresses. From our analysis, PARP was up-regulated in response to DTHT stress. In a previous study, down-regulation of PARP1 increased DT tolerance in Arabidopsis [72]. This suggests that up-regulation of PARP1 in response to DTHT in the switchgrass samples could reduce its DT tolerance.

### Genes deduced as HT responsive genes

As our primary focus in this experiment was on DT and DTHT responsive genes, we did not include HT only treatment. However, when we analyzed DTHT vs DT data for probable HT responsive genes, we found some interesting results. The HT responsive genes, i.e., HSPs that we detected are similar to HT genes found in wheat and switchgrass when exposed to only HT stress [16, 22]. The HT responsive genes identified in this experiment could serve as basis for future studies when imposing only HT stress.

### TFs responsive to individual DT and DTHT stress

The differential expression pattern of DT-responsive genes was accompanied by different families of TFs, including bHLH (basic helix-loop-helix), WRKY, NAC (NAM, ATAF and CUC) and ERF (ETHYLENE RESPONSE FACTOR). Transcription factors known to be involved in DT stress response include WRKY, C2H2 and NAC, and these were more abundant in DT compared to DTHT samples (as shown in the TF statistics in Additional File 10). This finding may suggest that these TFs were induced early to initiate a transcriptional response to DT stress. Interestingly, the TFs mentioned above were identified in Populus species (*Populus davidiana*) under DT stress [73]. The bHLH TF was identified to be more highly expressed in response to DT stress alone, compared to DTHT stress in switchgrass. Mun et al. identified a strong expression pattern of bHLH in *P. davidiana* at 6 h and 12 h time points of their study [73]. Also, PebHLH35 as one of the families of bHLH, has been recognized to play a significant role in DT tolerance by controlling stomatal development and photosynthesis in Arabidopsis [74]. TFs such as MYB, bHLH, and WRKY were also abundantly identified in *Brassica juncea* plants under DT stress [61]. A high number of MYB and CH3 TFs were identified in DTHT samples compared to DT samples. MYB TF is known to control various processes including development, metabolism, and responses to biotic and abiotic stresses. A previous report showed that AtMYB096 from Arabidopsis is associated with ABA and JA-mediated pathway and provided DT tolerance in Arabidopsis. In another study, BcMYB1 TF from *Boea crassifolia* is reported to provide DT tolerance [75]. There were relatively more NAC related TFs identified in response to DTHTstress (Additional file 10). However, some NAC TFs were either down-regulated or up-regulated, a differential expression of the TFs have been indicated in Additional file 9 For example, NAC domain-containing protein 47, NAC domain-containing protein 83, and NAC domain-containing protein 41 were down-regulated whereas NAC domain-containing protein 102 and NAC domain-containing protein were up-regulated. Various NAC genes have been studied in switchgrass. An example is an identification and functional characterization of PvSWNs in switchgrass. These NAC genes have been reported to be associated with lignin and biosynthetic pathway [76]. Various ERF (ethylene-responsive factor family) TFs were responsive to single DT stress and DTHT stress from our analysis (Table 1). ERF TF family has been characterized in a previous study, and they have been found to respond to HT stress in *Populus simonii* [64]. Similarly, ERF isolated from soybean (*GmERF7*) was induced by DT and salt stress. However, *GmERF7* was reported to be down-regulated during cold stress in the same study by Zhai et al. (2013) [77]. In both DT and DTHT responsive TFs, bHLH TFs had the highest number. In a previous study, bHLH TFs have been reported to be related to DT [74, 78, 79]. Other stress-responsive TF families such as WRKY, MYB, and NAC previously reported were identified [80]. After bHLH, the next highest TF family identified from the analysis is NAC (NAM, ATAF1,2, and CUC2). NAC is one of the largest TFs and has been shown as an important regulator of abiotic stresses [81, 82]. Reports indicate that NAC regulates DT stress when overexpressed in plants. Similarly, NAC genes, when overexpressed in Arabidopsis (*ANAC019*, *ANAC055,* and *ANAC072*) and rice (*OsNAC5, OsNAC6, OsNAC10*) enhanced DT and salt tolerance [83–85]. We also identified a high amount of bZIP TF encoding genes in both DT and DTHT samples. Similar to bHLH and NAC, bZIP TF family has been reported to respond to various abiotic stresses. In rice, bZIP has been related to DT with OsbZIP16 being listed as a key candidate gene for DT tolerance [86]. Interestingly, more C3H TF was induced during DTHT stress compared to only DT stress. Our study reveals C3H as a candidate TF for both DTHT tolerance studies in plants. Analysis of C3H TF family in *Aegilops tauschii* suggested that overexpression of *AetTZF1* caused the plant to be more tolerant to DT stress [87].

### Effect of DT and HT stress on phenylpropanoid metabolism

Phenylpropanoid is associated with lignin or flavonoid biosynthesis and plays essential role in the production of quality feedstock. Although phenylpropanoid pathway was not identified from the KEGG pathway or GO analysis, genes that are involved in the phenylpropanoid pathway previously identified by Ayyappan et al. [88] such as cinnamate-4-hydroxylase (C4H) with gene ID Pavir.J661300.v4.1, hydroxycinnamoyl-CoA shikimate/quinate hydroxycinnamoyltransferase (HCT) (gene ID Pavir.6KG280500.v4.1) were down-regulated with an extreme temperature at time point 168/96 h. Except for cinnamyl alcohol dehydrogenase 9 (CAD9) (Pavir.7NG065100.v4.1), which was up-regulated (Additional file 5). The role of CAD9 in lignin composition have been reported by Kim et al. [89]. CAD9 has been reported to catalyze the final step required to complete the production of lignin monomers such as coniferyl alcohol, sinapyl alcohol, and 4-coumaryl alcohol [90]. The presence of lignin limits the bioconversion of carbohydrates to ethanol from switchgrass. This limitation can lead to the high cost of cellulosic ethanol production; therefore, an effective approach previously reported was to cause down-regulation of the genes involved in lignin biosynthesis to reduce lignin production [91, 92]. From our analysis, CAD9 was found to be up-regulated in the DT and HT-treated samples. This finding suggests that DT and HT stress could cause an increase in lignin synthesis. Lignin biosynthesis negatively correlates with biomass and bioenergy production in switchgrass because of the recalcitrant nature of the cell wall [93]. In another study, down-regulation of the CAD gene in switchgrass by RNA silencing led to a reduction in the amount of lignin and increased biomass production [76]. We observed down-regulation of phenylpropanoid genes, HCT, and C4H. Down-regulation of HCT and C4H could be due to the general down-regulation of genes involved in metabolism in response to stresses. These genes can serve as a target for genetic manipulation to produce quality biomass in switchgrass.

In addition to regulating development, differentiation, metabolism, biotic and abiotic processes, TFs belonging to MYB proteins have been found to play a significant role in phenylpropanoid metabolism [75]. From our analysis, several MYB TFs were responsive to DTHT compared to the individual DT stress. The transcript Pavir.6KG070500.v4.1 which is annotated as a MYB-related family protein, was significantly down-regulated at three different time points from the analysis. MYB proteins also serve to regulate other branches of phenylpropanoid metabolism. TF AmMYB305 from *Antirrhinum majus*, and MYB from Arabidopsis have been identified with a function in phenylpropanoid metabolism [94, 95]. Switchgrass R2R3-MYB (PvMYB4) TF has been identified and characterized. PvMYB4 is reported to bind to AC-I, AC-II and AC-III elements of the monolignol pathway causing down-regulation of the genes in vivo. PvMYB4 is known to suppress phenylpropanoid metabolism and the quantity of lignin in switchgrass and tobacco. Overexpression of PvMYB4 caused a reduction in the lignin content and decreased recalcitrance in transgenic switchgrass [96]. Hence, down-regulation of MYB related proteins from our analysis during DTHT stress may increase lignin production to affect biomass and biofuel production in switchgrass. This finding suggests that the MYB transcription factor should be considered in enhancing biomass under DT and extreme temperature conditions.

### Validation of differentially regulated genes

We selected seven genes from the list of significantly regulated genes to validate experimentally by performing RT-PCR and qPCR. Five of the selected transcripts were either down or up-regulated in response to combined DT and HT stress. These transcripts include Pavir.3KG247300.v4.1, Pavir.9KG154500.v4.1, Pavir.9KG545000.v4.1, Pavir.4KG077400.v4.1, and Pavir.4KG264600.v4, which were annotated as a copper amine oxide, ATP dependent protease, UB-like protease 1A, leucine-rich receptor-like protein, and phosphatidylethanolamine-binding protein respectively. Copper amine oxide and UB-like Protease 1A were up-regulated in response to DT and HT stress while ATP-dependent protease, the leucine-rich receptor-like protein, was down-regulated in response to DTHT stress. Another transcript Pavir.6KG130600.v4.1 which is annotated as UDP-glucosyl transferase 85A3 was up-regulated and down-regulated at different time points in response to single DT stress as indicated in Fig. 6b. UDP-glucosyltransferase 85A3 from switchgrass was down-regulated with severe DT at DT-168 h. A UDP-glycosyltransferase 76C2 (UGT76C2) belonging to the same family as UGT85A played a significant role in response to water deficit in a previous report Arabidopsis. Like our finding UGT76C2 from Arabidopsis was down-regulated in response to DT stress [97].

Our analysis found that the transcript Pavir.9NG755000.v4.1 which is annotated as basic chitinase, was only identified in samples exposed to DT and HT switchgrass samples. This gene was down-regulated in all the time points but was significantly up-regulated at extreme DT and HT (Additional file 5). RT-PCR confirmed results from the RNA-Seq data, and which is consistent with the previous report on the function of chitinase genes (figure not shown). Chitinase enzymes are reported as defense proteins and their expression are usually influenced by environmental stress [98]. They provide resistance against pathogens and is tolerant to various environmental stresses. Chitinase genes have been recognized to respond to environmental stresses. In a previous study, the expression of one of the chitinase enzymes was enhanced in Arabidopsis samples with allosamidin and strong HT stress compared to control plants [99]. Similar to our findings, Pavir.9NG755000.v4.1 annotated as chitinase may have been differentially expressed due to the HT stress. The up-regulation of the chitinase gene may help to improve DT and HT stress tolerance in switchgrass.

## Conclusion and future perspectives

Several studies have been conducted in switchgrass in response to individual biotic or abiotic stress. However, scientific information on the transcriptional changes in switchgrass under combined DT and HT stress is underexplored. We utilized RNA-Seq approaches to elucidate transcriptomic changes in switchgrass when exposed to either DT or a combination of DT and HT. Many of the genes identified were in response to DTHT stress. Additionally, we identified TFs that were regulated by these stresses. We found an overlap of genes in response to a single DT and a combination of DTHT stress. Interestingly, these transcripts were found in the droughtDB. Both single DT and DTHT had an effect on the photosynthetic machinery and produced genes involved in oxidative stress damage which can affect biomass production. Several HSPs and chaperones were produced in the combined DT and HT switchgrass samples compared to those with individual DT stress. The GO annotation and KEGG pathway analysis showed connections between the identified GO terms. Genes associated with the photosynthesis machinery and control carbon fixation were down-regulated, suggesting the effect of DTHT on biomass production. A co-expression analysis revealed a unique expression pattern of the differentially expressed genes, which were classified into modules. Moreover, the significant pathways enriched in most of the DEG genes were involved in the metabolic and ABA signaling pathways.

Further, the combined DT and HT stress resulted in a unique regulation of genes and TFs involved in the phenylpropanoid pathways such as CAD9, C4H and HCT. CAD9, C4H and HCT are associated with lignin biosynthesis, which negatively correlates with biomass and bioenergy production. The stress-responsive genes and TFs identified in this study will be helpful in developing switchgrass cultivars with improved tolerance to DT and HT stress. The transcriptome data generated in this study could be used as a reference to investigate further DT and HT stress tolerance in bioenergy crops and plants in general.

## Materials and methods

### Growth and treatment of plants

**Fig. 8:**
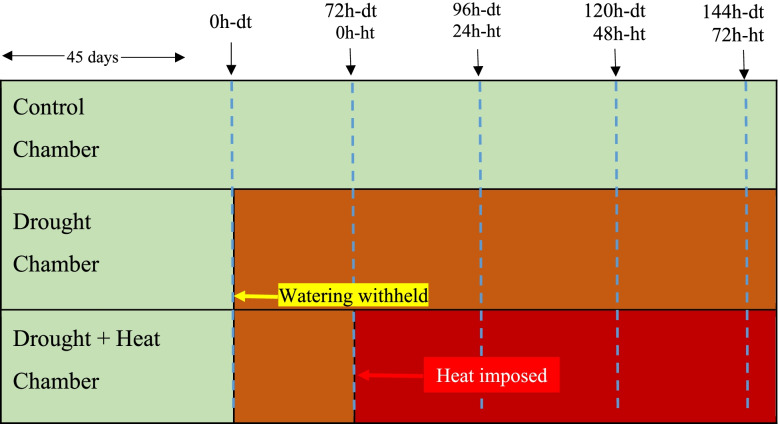
Control chamber: Regular watering (80% FC) and optimum temperature (30°/23 °C day/night temperature); DT chamber: withhold watering at 45 days after transplanting the ramets and kept at optimum temperature (30°/23 °C day/night temperature); DT + HT chamber: imposed HT after 72 h of DT (35°/25 °C day/night temperature); Leaf tissue samples were collected at 0 h-DT (dt), 72 h-dt/0 h-HT (ht), 96 h-dt/24 h-ht, 120 h-dt, 48 h-ht, and 144 h-dt/72 h-ht impositions

The experiments were conducted using a lowland ecotype Alamo, AP13 genotype. The AP13 genotype was a selection from the publicly available switchgrass cultivar ‘Alamo’. Initial selection was made at the University of Georgia, but later the genotype was moved to the greenhouse at Noble Research Institute, LLC. Clonal copies of the genotypes have been maintained in Noble greenhouse. Ramets of AP13 were transplanted into 3GP nursery pots (Growers Solution, Cookeville, TN) and grown for 40 days under optimum growing condition in the greenhouse and transferred to growth chambers at the Noble Research Institute, Ardmore, OK. The experiment was designed to mimic conditions in the natural environment where plants experience more than one type of stress. The goal is to identify the unique response of switchgrass to combined DTHT stress. The experiment was started 5 days after transfer to the growth chamber. The experiment was laid out in a randomized complete block design with three biological replicates. Six pots were assigned to control, 9 pots to DT, and 9 pots to DT and HT treatments at random during the transfer. The pots assigned to the three treatments were divided into three groups and assigned to the three replicates at random. The control and DT treatments were transferred to a growth chamber and the DT imposed with HT treatment was arranged in another growth chamber at random (Fig. 8). Leaf tissue samples were collected as indicated in Fig. 8 at the same time (starting at 2:00 PM) of the day for all samples collected. Plant tissues were immediately frozen in liquid nitrogen and stored at − 80 °C. The samples were then shipped to Delaware State University on dry ice overnight. Soil moisture at 10 cm depth of the pot was measured concomitant with tissue sample collection using FieldScout TDR 100 Soil Moisture Meter (Spectrum Technologies, Aurora, IL). Leaf SPAD reading was also taken at the same time. A diagram to indicate how the growth chamber was separated for DT and HT treatments have been shown in Fig. 8.

### RNA isolation and cDNA synthesis

Total RNA was extracted from leaves of control, DT, HT, and combined DT and HT-treated switchgrass using RNeasy Plant mini kit (Qiagen Inc., CA) according to the manufacturer’s instruction. To eliminate contaminating genomic DNA, all RNA samples were treated with amplification grade DNase I (Invitrogen) following manufacturer’s protocol. The concentration and purity of the RNA samples were determined using Nanodrop 2000 spectrophotometer (Thermo Scientific, Wilmington, DE). The A260/A280 nm ratios for a majority of the samples were 2.1. The quality of the RNA samples was determined by 1% agarose gel electrophoresis and Bioanalyzer 2100 (Agilent Technologies, Santa Clara, CA) for 28S/18S rRNA band intensity (2:1) and RNA integrity number (RIN) > 8. The RNA samples were stored at − 80 °C for use in downstream experiments. 1 μg of DNase treated RNA was used for cDNA synthesis using Protoscript II First Strand cDNA Synthesis kit (New England Biolabs, Ipswich MA) following the manufacturer’s instructions. In synthesizing the complementary DNA, 1 μg of DNase treated RNA was denatured with Oligo dT at 65 °C for 5 min; followed by adding Protscript II reaction mix and Protoscript II enzyme mix which were incubated at 42 °C for 60 mins. The Protoscript II enzyme was denatured at 80 °C for 5 mins and the cDNA was then stored at − 20 °C.

### Library construction and sequencing

A Fragment Analyzer (Advanced Analytical, Ames, IA) was used to check the quality and purity of all the RNA samples. RNA-Seq libraries were prepared using Illumina TruSeq Stranded mRNA Sample Preparation Kit (Illumina Inc., San Diego, CA) following the manufacturer’s instructions at the Delaware Biotechnology Institute, Newark, DE, USA. The libraries were sequenced on Illumina HiSeq 2500 platform with 101 bp paired-end reads.

### Processing of RNA-Seq data

FASTX-Toolkit (http://hannonlab.cshl.edu/fastx_toolkit/; v0.0.14) was used to perform quality control for RNA-Seq data requiring at least a 30 base quality score and at least 50 bps of read length. TopHat (v2.1.1) [100] was then used to align the reads to the switchgrass reference genome (Additonal file 1). FPKM values were calculated using the Cufflinks (v2.2.1) suite of tools [101]. To get the read count for the genes, HTSeq (v0.7.0) was used [102].

### Filtration of genes based on FPKM values

Low-expressed features tend to reflect noise and correlations based on counts that are mostly zero and are not meaningful. Based on the annotation file released, there are 91,838 genes across the switchgrass genome. FPKM values for each gene in each sample were calculated using cuffnorm in the Cufflinks suite of tools [101]. A given gene is retained for further analysis if at least half of the 15 groups have average FPKM value > 1 and the average FPKM value of all samples included is > 1 [103]. In total, 32,190 genes were retained for downstream analysis.

### Identification of DT and HT responsive genes

To identify DT responsive genes in the RNA-Seq samples, DESeq2 package was used [104]. First, genes that were differentially expressed between DT treatment group and control group at 0 h were excluded. Then the remaining genes that were differentially expressed between DT treatment group and control group in at least one of the following time points (72, 96, 120, 144 or 168 h) were defined as DT responsive genes. To identify responsive genes related to combination of DT and HT (DTHT), genes that were differentially expressed between group with combination of DT and HT treatment and control group at 0 h and 72 h were excluded. Then the remaining genes that were differentially expressed between group with combination of DT and HT treatment and control group in at least one of the following time points (96, 120, 144 or 168 h) were defined as DTHT responsive genes. Although the switchgrass plants were not exposed to direct heat temperatures separately, an assumption was made that the DEGs in the combined DTHT vs DT samples could be due to the heat stress imposed. To identify responsive genes that may be related to HT stress, genes that were differentially expressed between group with combination of DTHT treatment and DT group at 0 h and 72 h were excluded. Then the remaining genes that were differentially expressed between group with combination of DT and HT treatment and DT treatment group in at least one of the following time points (96, 120, 144 or 168 h) were defined as HT responsive genes.

### Construction of co-expression network using WGCNA

Log2 transformed FPKM matrix of the genes (32,190) was used as input to WGCNA (v1.51) (Additional file 4). The function “pickSoftThreshold” was used to pick an approximate power value. Then “blockwiseModules” (networkType = “signed hybrid”) was used to construct co-expression network.

### Functional analysis of stress responsive genes

#### GO enrichment analysis

For stress responsive genes or the genes in the co-expression networks, the corresponding GO terms of the genes were extracted. Singular Enrichment Analysis (SEA) from agriGO [105] was used to perform GO enrichment analysis.

#### KEGG enrichment analysis

For stress responsive genes, the corresponding KEGG orthology terms of the genes were also extracted. ClusterProfiler (v 3.0.5) [106] were then used to perform KEGG enrichment analysis.

#### MapMan analysis

To further understand the biological functions of the DEGs and specific pathways or genes associated with single DT and combined DTHT samples, we conducted metabolic pathways analysis using the MapMan software (http://MapMan.gabipd.org).

Default settings in MapMan software do not support mapping for the switchgrass genome. A customized input file was created using the Mercator [107] tool and protein sequences from switchgrass v4.1. The Mercator is a tool to batch classify protein or gene sequences into MapMan functional plant categories and create a draft metabolic network which can be directly used in MapMan software. Mercator output was used as mapping file for MapMan.

#### Annotation of transcription factor

Genome-wide identification of TF were performed using PlantTFDB 4.0 [108]. Proteins of primary transcript for the genes were uploaded to the prediction server of PlantTFDB 4.0. The output of the prediction severs included TF types and best hits in Arabidopsis.

### Quantitative real-time (qRT-PCR) analysis

QRT-PCR was performed using the synthesized cDNA. The primers were designed based on the differentially expressed transcripts of DT and combined DT and HT stresses (DTHT). These primers will be used to validate the quantitative expression of the genes with highly expressed transcripts (log2FC > 2) from DTHT analysis. The selected DTHT and DT genes and the list of specific primer sequences are given in (Additional file 14)). The primers were designed using the online tool for real-time PCR (TaqMan) primer design by GenScriptUSA Inc. (Piscataway, NJ). A conventional PCR was first performed to validate the primers before using them in qRT-PCR. One microliter of fifty nanogram of cDNA was used a template for the conventional PCR reaction under these conditions (95 °C for 1 min, 55 °C for 30 s and 72 °C for 1 min) for 35 cycles. The PCR product was separated on a 1% agarose gel stained with ethidium bromide.

qRT-PCR was performed using an ABI 7500 real-time PCR system and SYBR Green Kit (Applied Biosystems, Grand Island, USA). Twenty-five μLs of the PCR reactions containing1 μg of 1st-strand cDNA, 12.5 μL of Power SYBR Green Master Mix, and 3 μL of 10 nM specific primers (forward and reverse) and 9.5 μL of water. The reference gene *Actin11* was used as an internal control primer to normalize the results in all the samples. The PCR conditions for the qRT-PCR were the following; 95 °C for 10 min, followed by 40 cycles of 95 °C for 15 s and 65 °C for 1 min. The efficiency of the primers was tested, and the relative expression was determined from three biological and three technical replicates using ΔΔCT method (Schmittgen and Livak, 2010). Minitab-17 software (State College, PA) was used to analyze the normalized CT values from the qRT-PCR analysis.

### Availability of data and materials

The datasets supporting the conclusions of this research article have been included in the article and as additional files. The sequencing database for switchgrass under DT and HT stress has been deposited at NCBI under GEO accession number (GSE174278) https://www.ncbi.nlm.nih.gov/geo/query/acc.cgi?acc=GSE174278 and it can be downloaded.

## Supplementary Information

**Additional file 1.** Summary statistics of the RNA-Seq reads.

**Additional file 2.** Heat maps for the WGCNA modules: The heat map modules are in this order, module 1, module 2, module 3, module 5, module 7, module 8, module 9, module 14, module 15, module 16, module 17 and module 25.

**Additional file 3.** Heat map of the 386 switchgrass genes that have best Arabidopsis hits in droughtDB.

**Additional file 4.** FPKM values of genes with WGCNA module numbers.

**Additional file 5.** List of DEGs with annotation.

**Additional file 6.** GO enrichment analysis using agriGO.

**Additional file 7.** KEGG enrichment analysis using clusterProfiler.

**Additional file 8.** List of TFs in switchgrass.

**Additional file 9.** List of DT, DTHT, and HT responsive TFs.

**Additional file 10.** Statistics of numbers of TF types in the responsive genes.

**Additional file 11.** Numbers of DT, DTHT, and HT responsive genes in WGCNA modules.

**Additional file 12.** GO enrichment of genes in co-expression modules.

**Additional file 13.** List of switchgrass genes that have best Arabidopsis hit in droughtDB.

**Additional file 14.** List of primers for qRT-PCR validation of DT and DTHT genes.

## Data Availability

All of the data and materials supporting our research findings are contained in the methods section of the manuscript. Details are provided in the attached additional files.

## Abbreviations

DT: Drought
DTHT: Drought and heat stress
HT: Heat
DEG: Differentially expressed genes
QRT-PCR: Quantitative real-time PCR
GO: Gene Ontology
KEGG: Kyoto Encyclopedia of Genes and Genome
ABA: Abscisic acid
