# Supplementary figures and images for: Global analysis of switchgrass (Panicum virgatum L.) transcriptomes in response to interactive effects of drought and heat stresses
Source: BMC Plant Biol. 2022 Mar 8;22:107. doi: 10.1186/s12870-022-03477-0 (PMC8903725; doi:10.1186/s12870-022-03477-0)

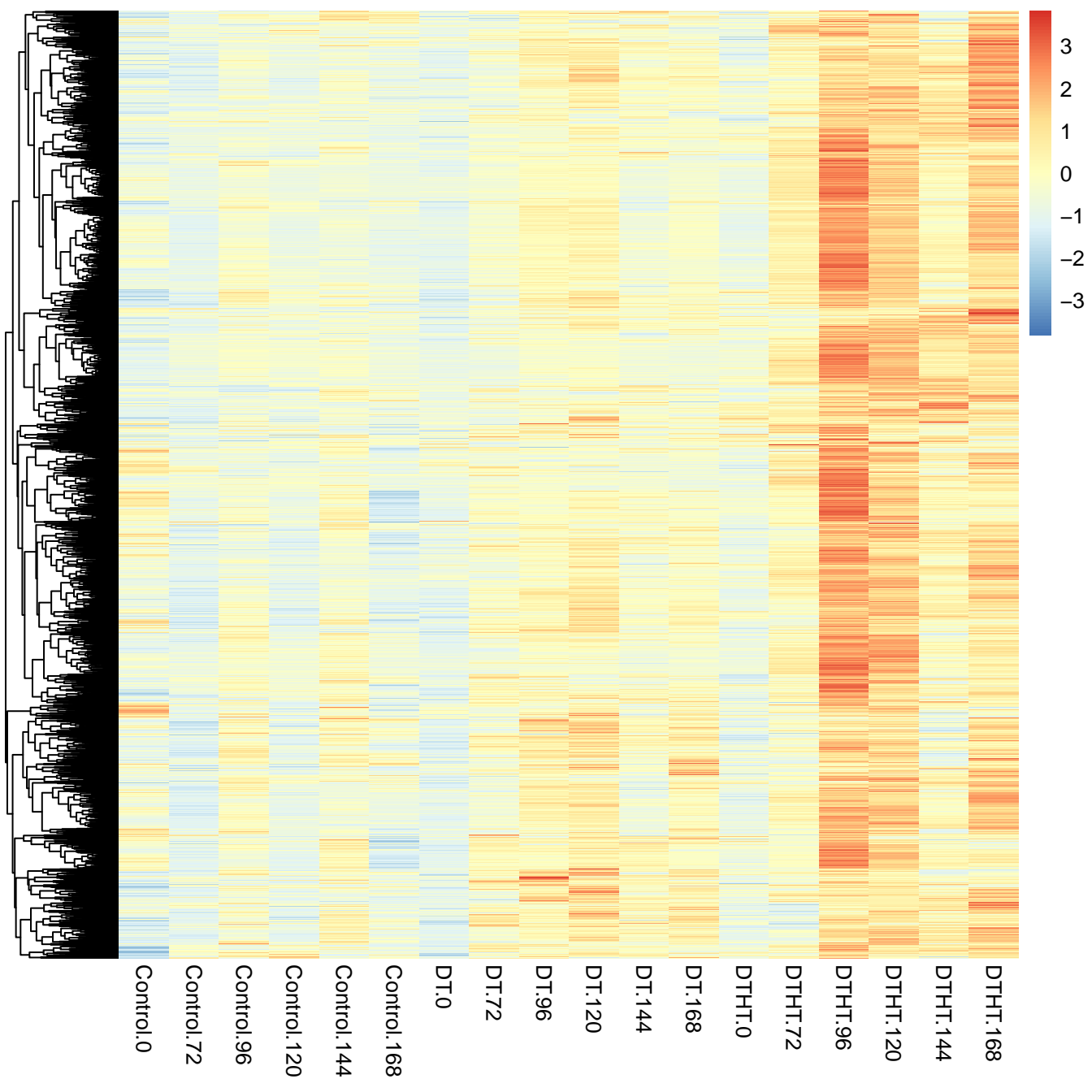

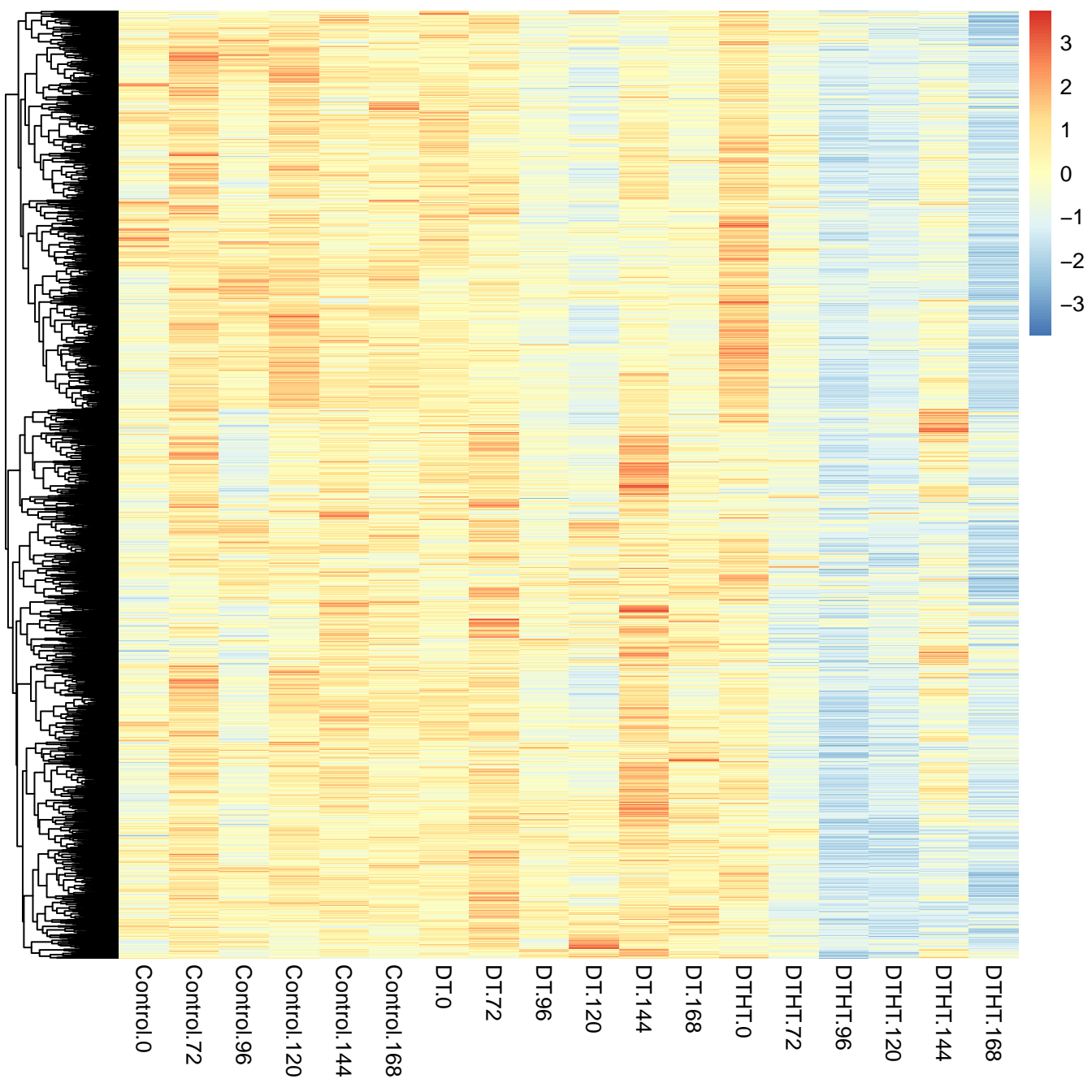

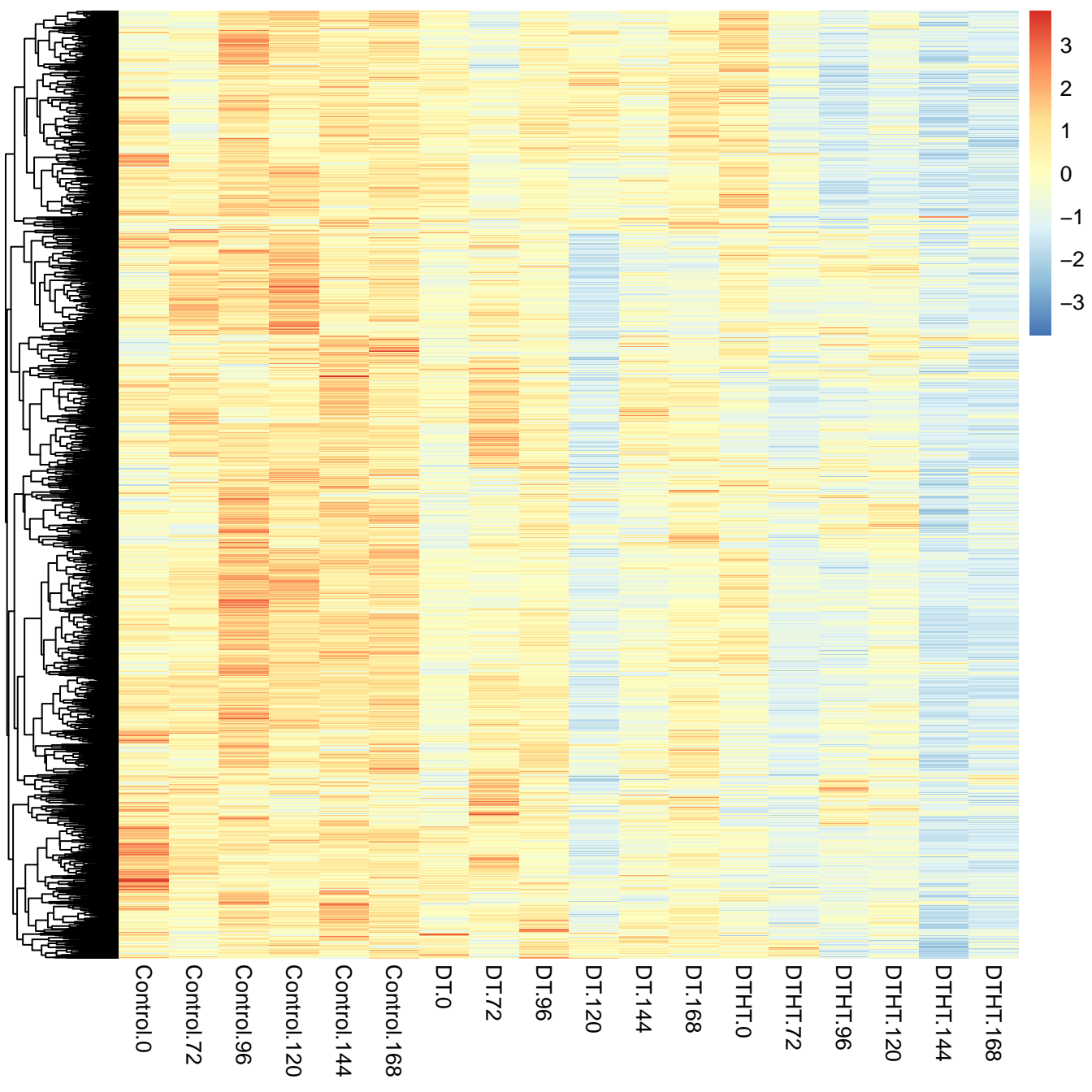

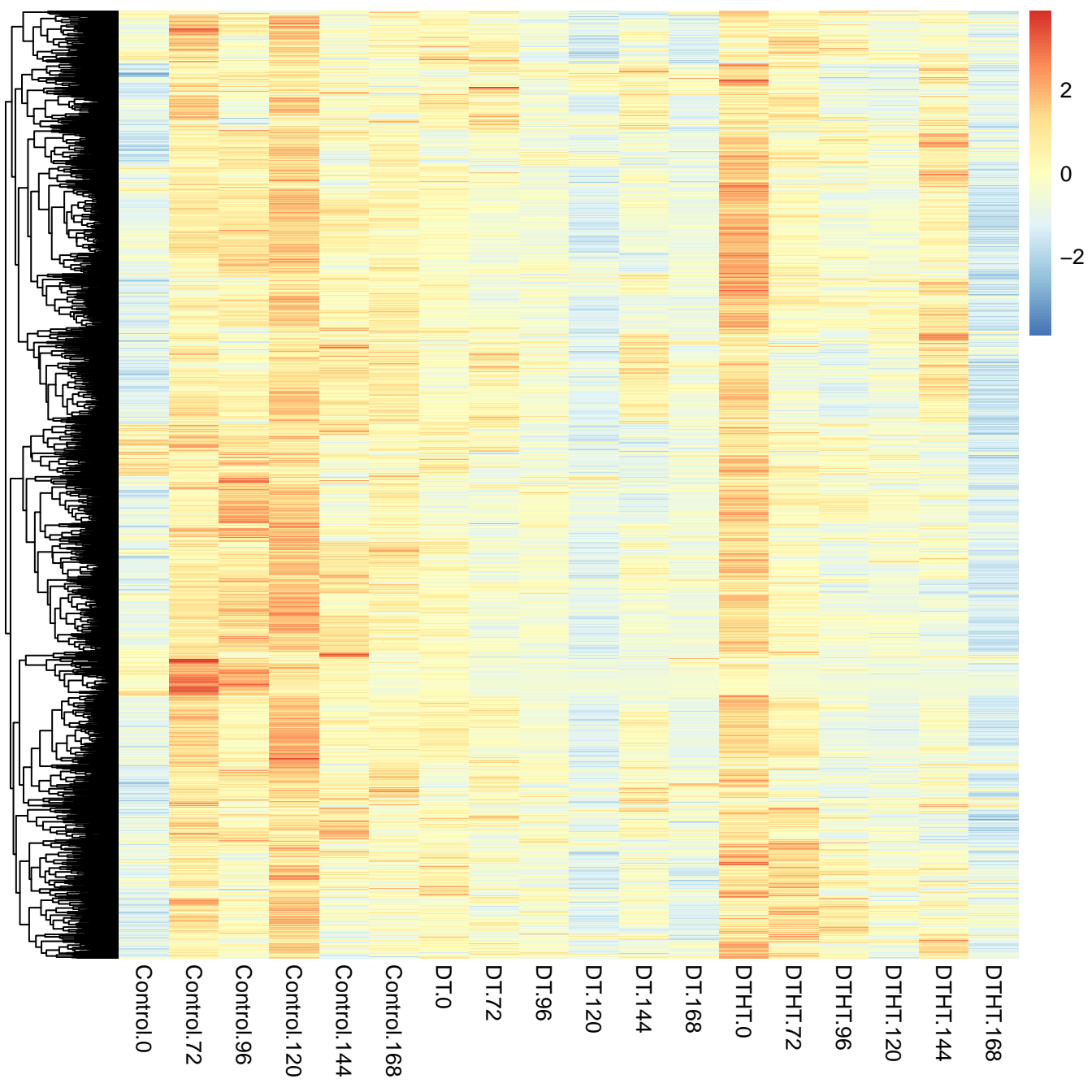

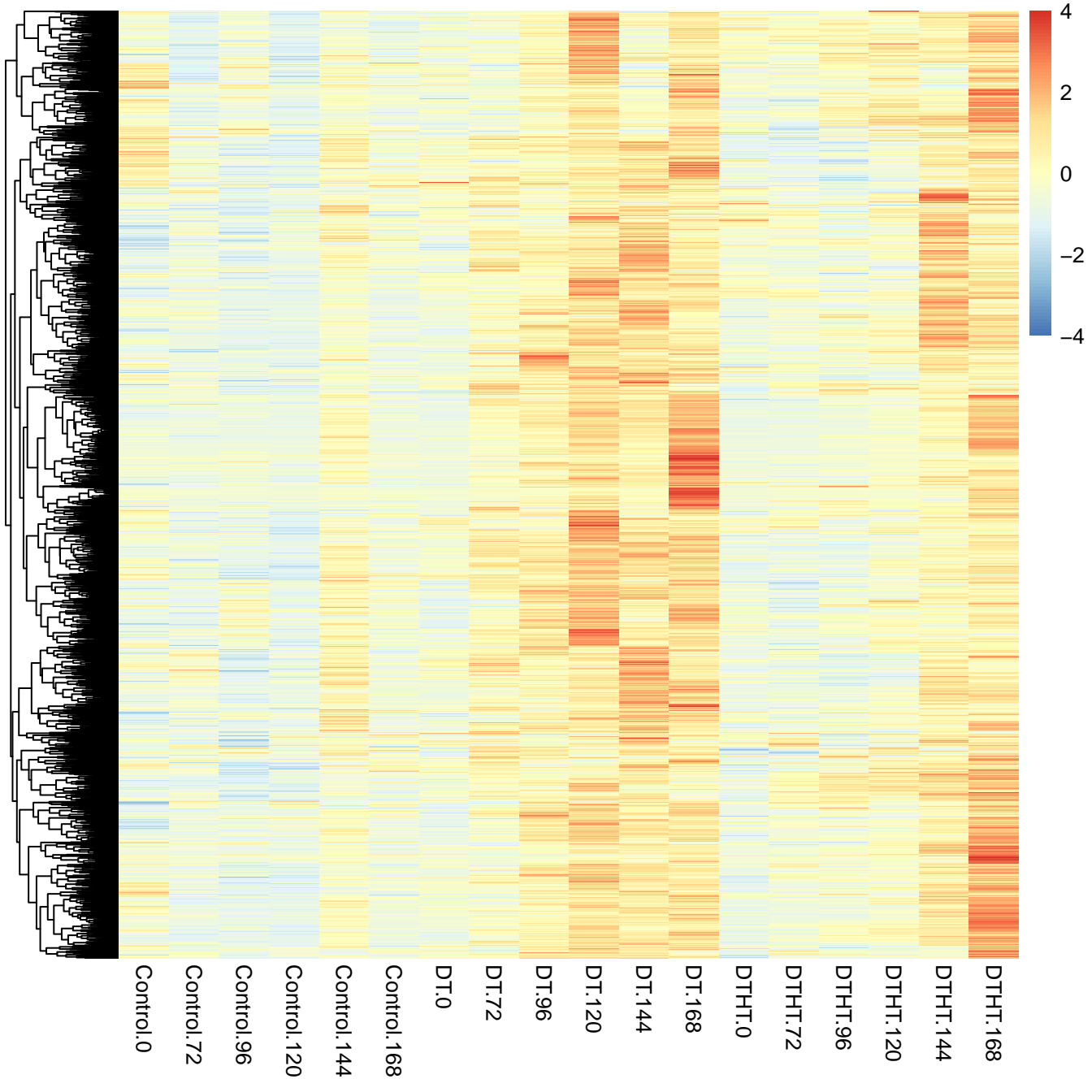

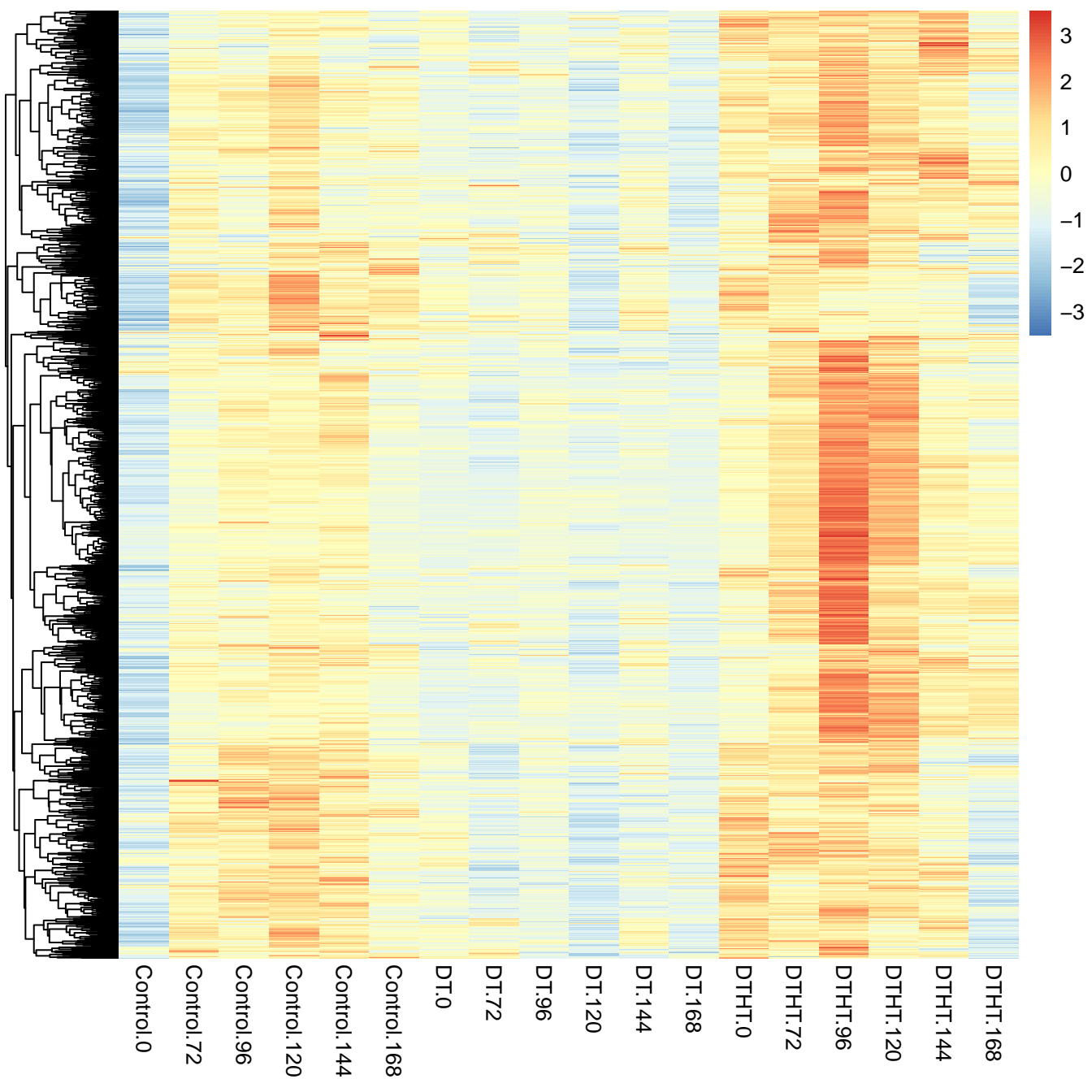

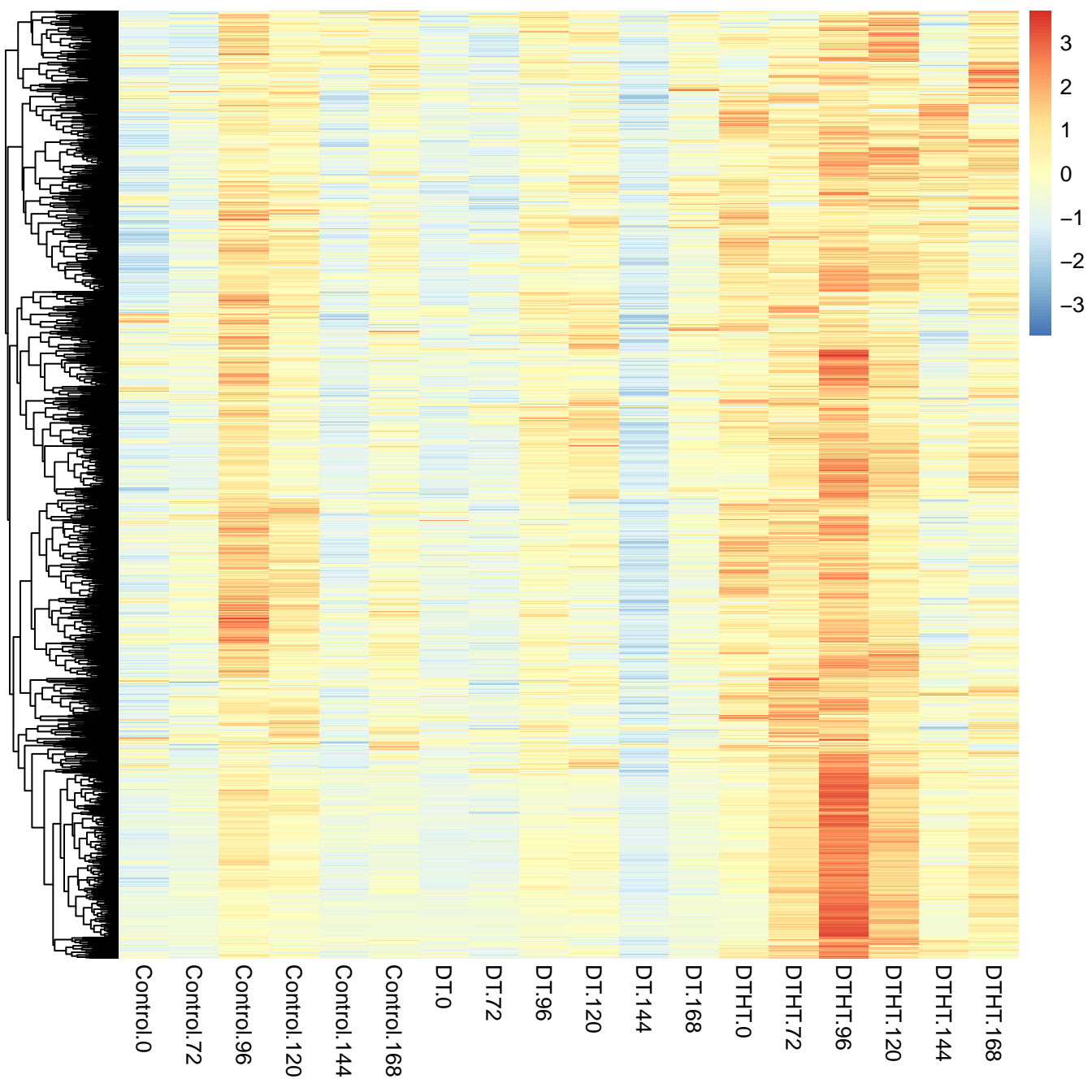

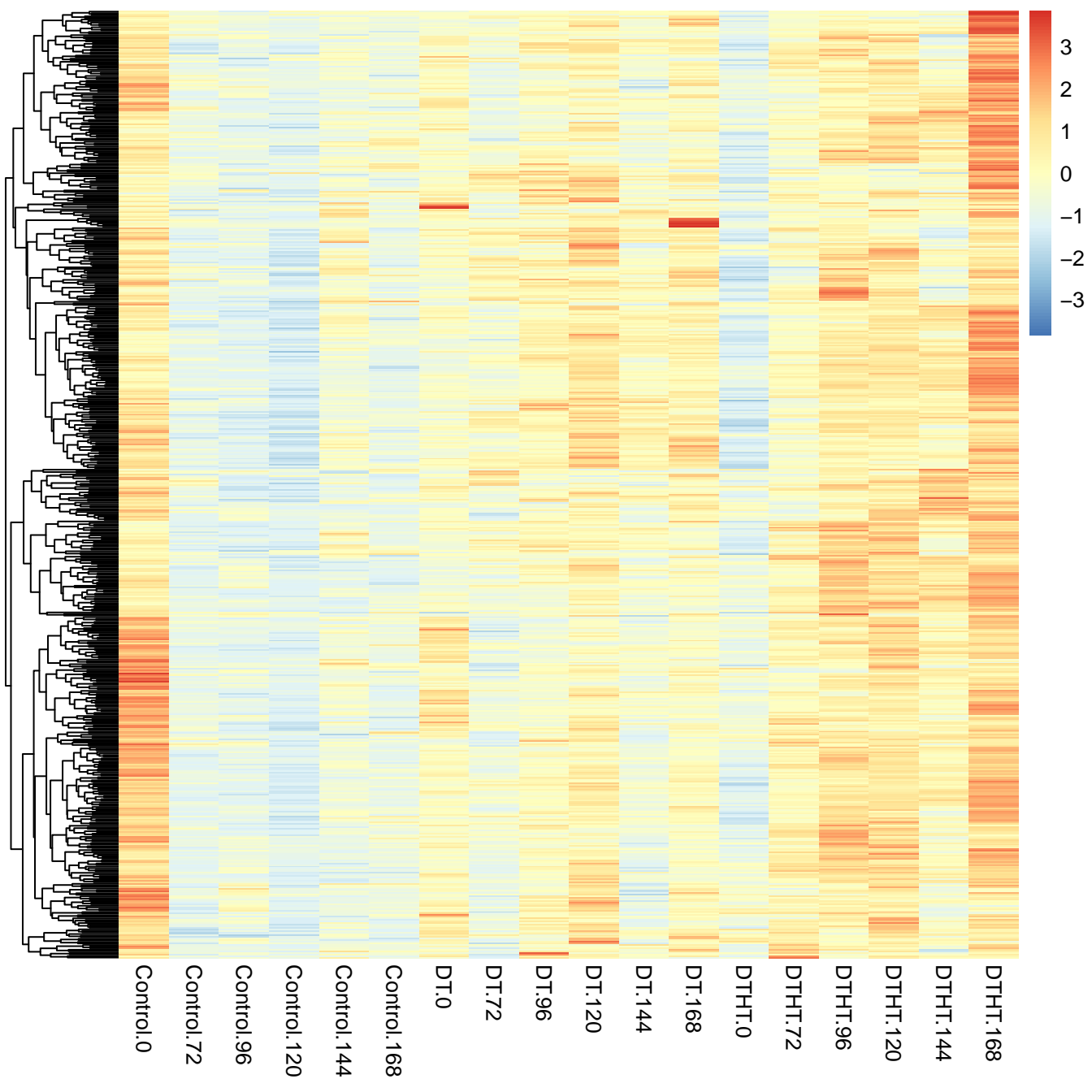

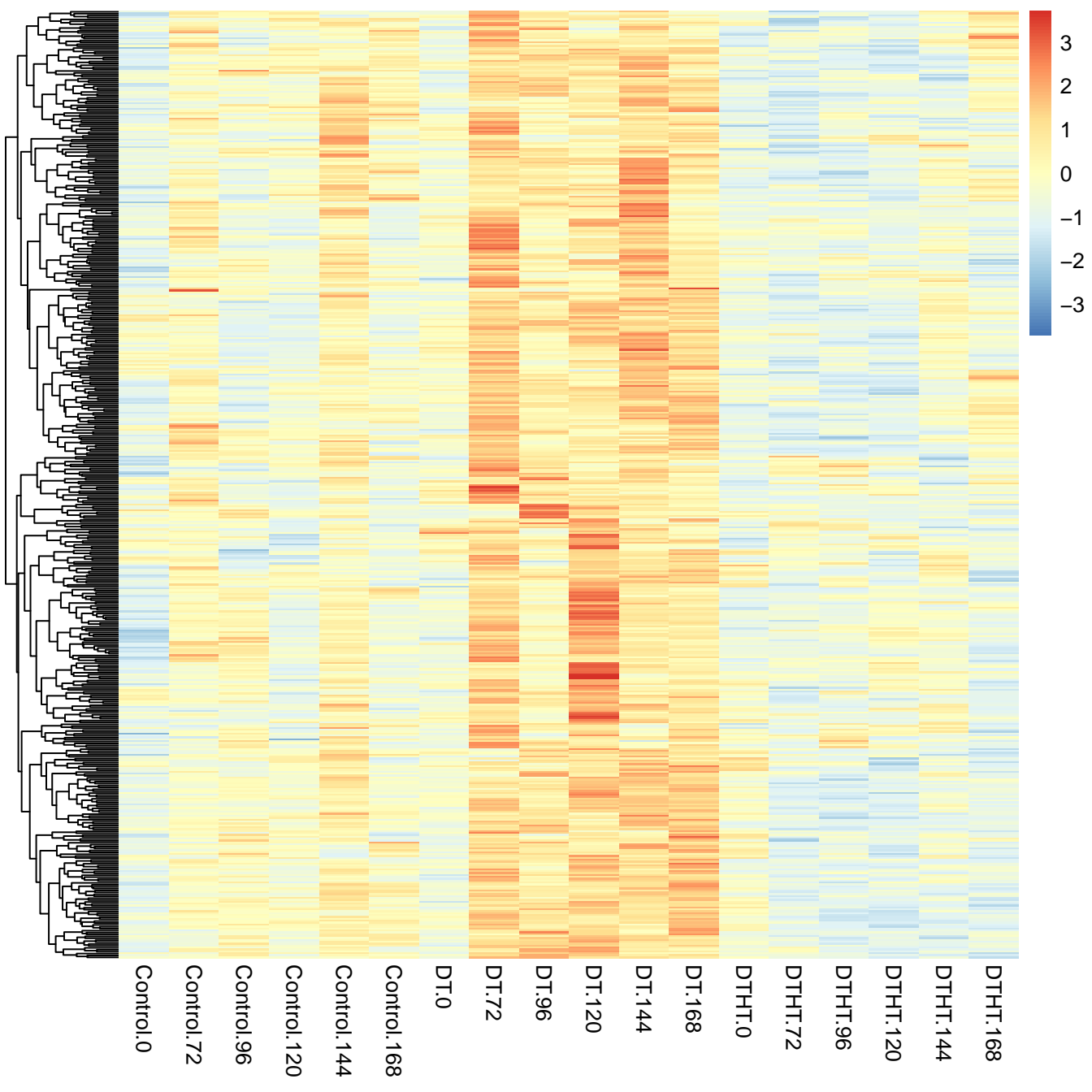

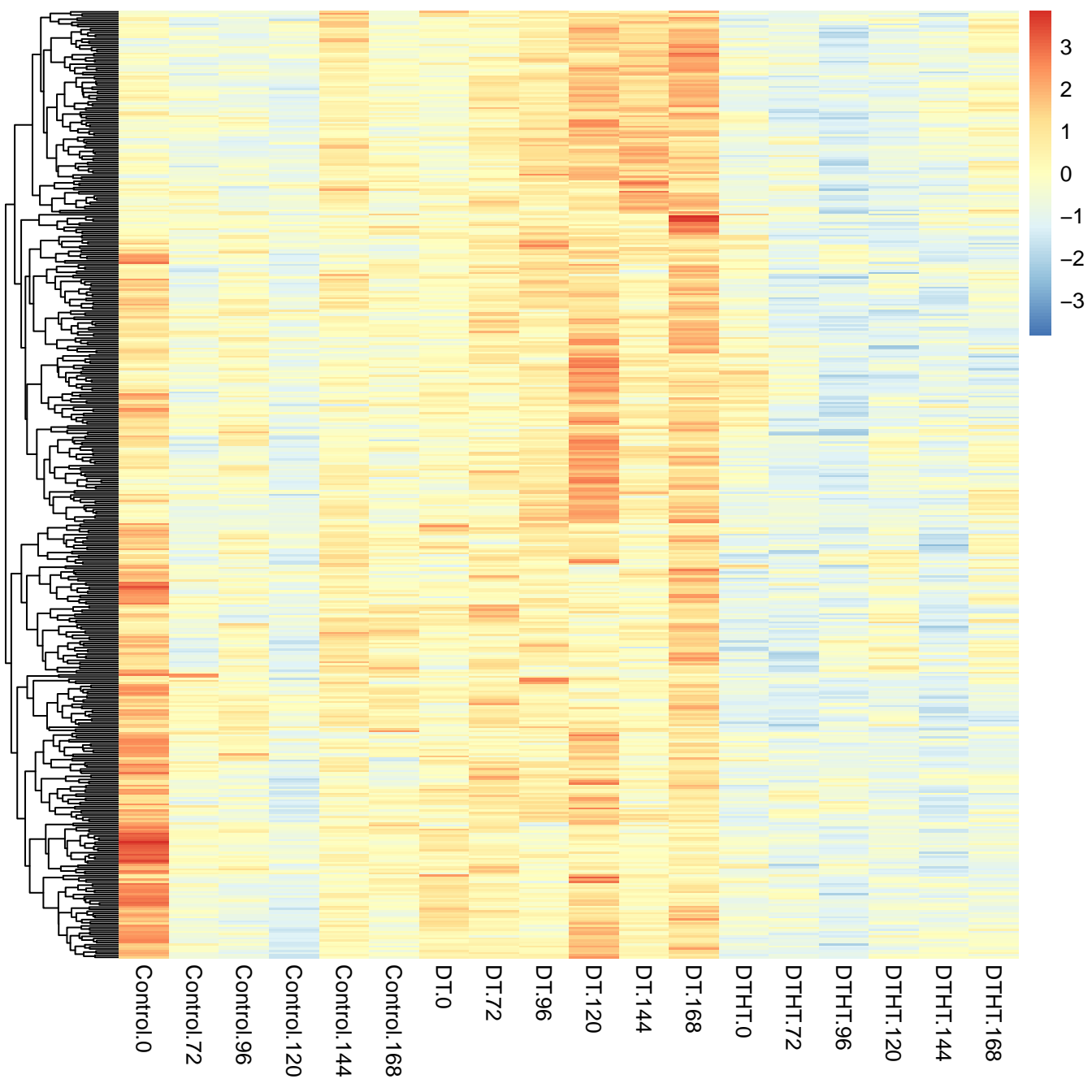

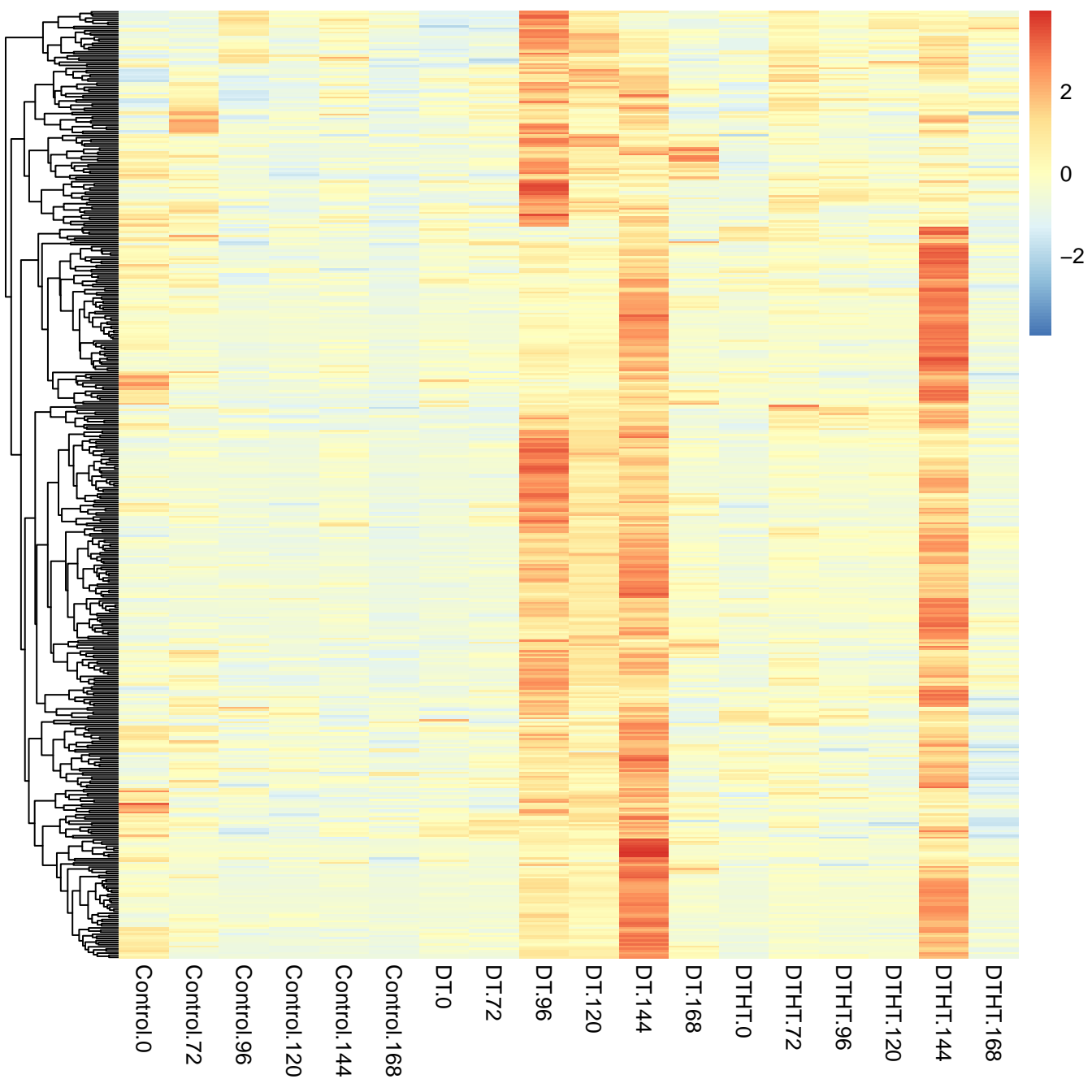

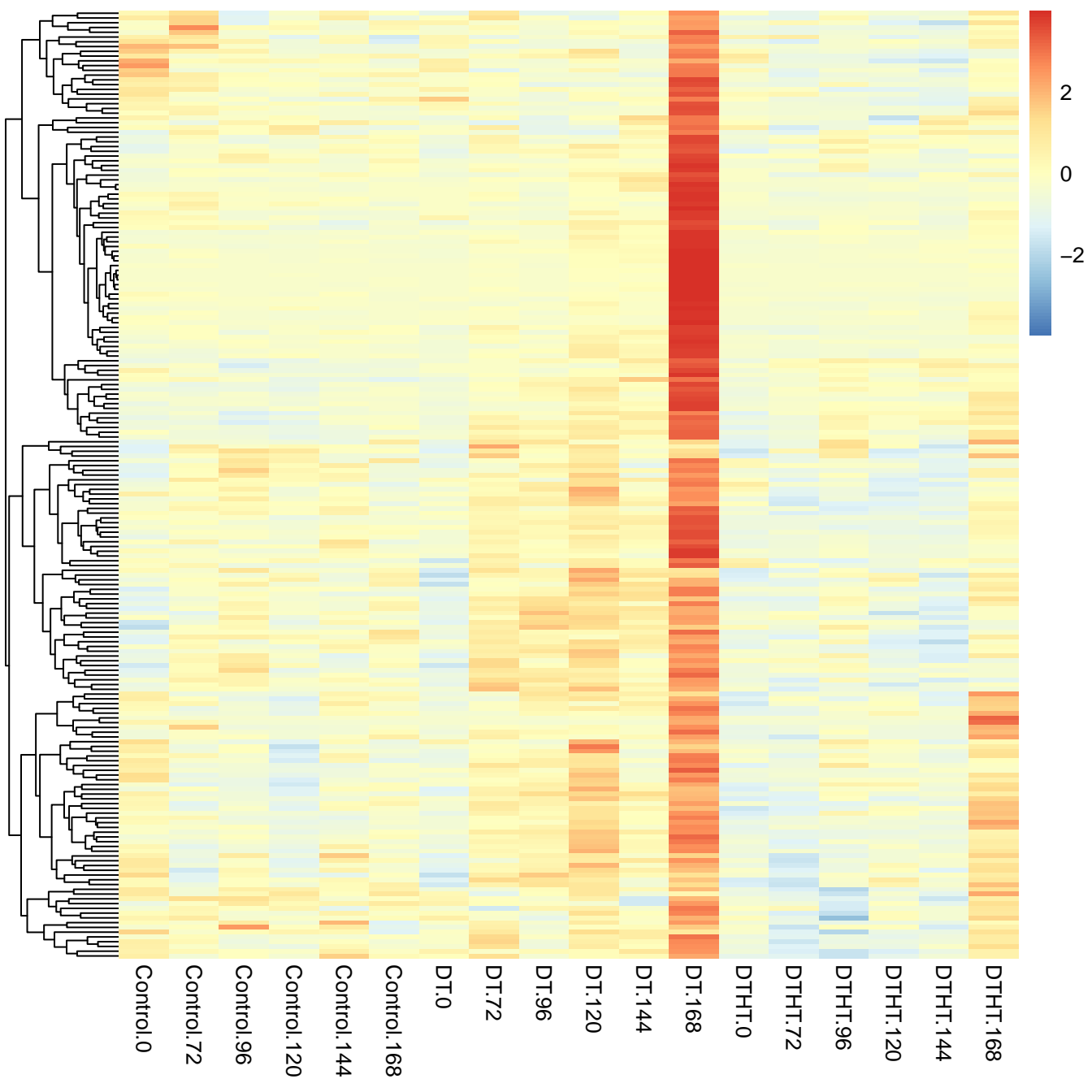

Supplement: Supplementary file 2 — Additional file 2. Heat maps for the WGCNA modules: The heat map modules are in this order, module 1, module 2, module 3, module 5, module 7, module 8, module 9, module 14, module 15, module 16, module 17 and module 25. [file 12870_2022_3477_MOESM2_ESM.pdf]

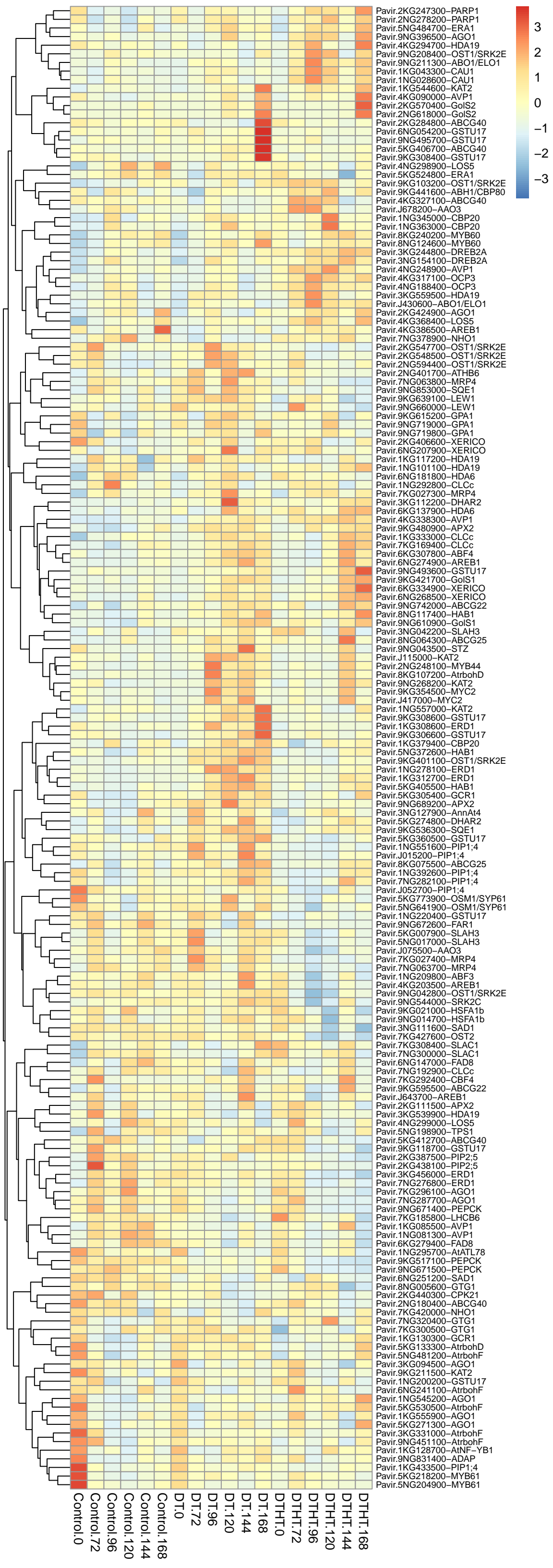

Supplement: Supplementary file 3 — Additional file 3. Heat map of the 386 switchgrass genes that have best Arabidopsis hits in droughtDB. [file 12870_2022_3477_MOESM3_ESM.pdf]
